# Reduced mitochondria membrane potential and lysosomal acidification are associated with decreased oligomeric Aβ degradation induced by hyperglycemia: A study of mixed glia cultures

**DOI:** 10.1371/journal.pone.0260966

**Published:** 2022-01-24

**Authors:** Yung-Cheng Huang, Shu-Meng Hsu, Feng-Shiun Shie, Young-Ji Shiao, Li-Jung Chao, Hui-Wen Chen, Heng-Hsiang Yao, Meng An Chien, Chung-Chih Lin, Huey-Jen Tsay

**Affiliations:** 1 Department of Physical Medicine and Rehabilitation, Cheng-Hsin General Hospital, Taipei, Taiwan, Republic of China; 2 National Taipei University of Nursing and Health Sciences, Taipei City, Taiwan, R.O.C; 3 Institute of Neuroscience, School of Life Science, National Yang Ming Chiao Tung University, Taipei, Taiwan, R.O.C; 4 Center for Neuropsychiatric Research National Health Research Institutes, Zhunan Town, Miaoli County, Taiwan, R.O.C; 5 National Research Institute of Chinese Medicine, Ministry of Health and Welfare, Taipei, Taiwan; 6 Ph.D. Program in Clinical Drug Development of Chinese Herbal Medicine, Taipei Medical University, Taipei, Taiwan, R.O.C; 7 Institute of Biopharmaceutical Science, National Yang Ming Chiao Tung University, Taipei, Taiwan, R.O.C; 8 Department of Life Sciences and Institute of Genome Sciences, National Yang Ming Chiao Tung University, Taipei, Taiwan, Republic of China; 9 Brain Research Center, National Yang Ming Chiao Tung University, Taipei, Taiwan, Republic of China; 10 Biophotonics Interdisciplinary Research Center, National Yang Ming Chiao Tung University, Taipei, Taiwan, Republic of China; Nathan S Kline Institute, UNITED STATES

## Abstract

Diabetes is a risk factor for Alzheimer’s disease (AD), a chronic neurodegenerative disease. We and others have shown prediabetes, including hyperglycemia and obesity induced by high fat and high sucrose diets, is associated with exacerbated amyloid beta (Aβ) accumulation and cognitive impairment in AD transgenic mice. However, whether hyperglycemia reduce glial clearance of oligomeric amyloid-β (oAβ), the most neurotoxic Aβ aggregate, remains unclear. Mixed glial cultures simulating the coexistence of astrocytes and microglia in the neural microenvironment were established to investigate glial clearance of oAβ under normoglycemia and chronic hyperglycemia. Ramified microglia and low IL-1β release were observed in mixed glia cultures. In contrast, amoeboid-like microglia and higher IL-1β release were observed in primary microglia cultures. APPswe/PS1dE9 transgenic mice are a commonly used AD mouse model. Microglia close to senile plaques in APPswe/PS1dE9 transgenic mice exposed to normoglycemia or chronic hyperglycemia exhibited an amoeboid-like morphology; other microglia were ramified. Therefore, mixed glia cultures reproduce the *in vivo* ramified microglial morphology. To investigate the impact of sustained high-glucose conditions on glial oAβ clearance, mixed glia were cultured in media containing 5.5 mM glucose (normal glucose, NG) or 25 mM glucose (high glucose, HG) for 16 days. Compared to NG, HG reduced the steady-state level of oAβ puncta internalized by microglia and astrocytes and decreased oAβ degradation kinetics. Furthermore, the lysosomal acidification and lysosomal hydrolysis activity of microglia and astrocytes were lower in HG with and without oAβ treatment than NG. Moreover, HG reduced mitochondrial membrane potential and ATP levels in mixed glia, which can lead to reduced lysosomal function. Overall, continuous high glucose reduces microglial and astrocytic ATP production and lysosome activity which may lead to decreased glial oAβ degradation. Our study reveals diabetes-induced hyperglycemia hinders glial oAβ clearance and contributes to oAβ accumulation in AD pathogenesis.

## Introduction

Alzheimer’s disease (AD) is an age-related neurodegenerative disease. Recently, AD is also recognized as a metabolic disease [1]. The accumulation of aggregated amyloid beta (Aβ) and neuroinflammation are one characteristic hallmark of AD. Among the various conformations of Aβ aggregates, oligomeric Aβ (oAβ) elicits the most synaptic toxicity [2, 3]. The levels of oAβ correlate with the degree of cognitive decline in patients with AD with mild cognitive impairment [4]. Sporadic and familial AD account for more than 95% and less than 5% of AD cases, respectively. In familial AD, accumulation of Aβ is mainly due to defective processing of mutant amyloid precursor protein [5]. In contrast, the clearance of Aβ is decreased in the brain of patients with sporadic AD compared to normal individuals [6].

Hyperglycemia and hyperlipidemia are hallmarks of Type II diabetes, a systematic metabolic disorder [7]. Long-term hyperglycemia and hyperlipidemia contribute to the diabetic cardiomyopathy by attenuating the downstream of insulin signaling through activating NFκB. Furthermore, the crosstalk of glucose and lipid mediates the diabetic kidney disease through modulating the activity of sterol regulatory element-binding transcription factor [8]. Epidemiologic studies showed that metabolic syndrome such as Type II diabetes is associated with an increased risk of sporadic AD [9, 10]. Obesity and insulin resistance are the core features of metabolic syndrome which is closely related to Type II diabetes and AD [11]. Although diabetic hyperglycemia significantly contributes to the pathogenesis of AD in patients and transgenic mouse models, the underlying mechanisms remain unclear [9, 12, 13].

The brain glucose level correlates with elevated fasting plasma glucose levels in patients with diabetes, chronically elevated neuronal glucose levels may alter the kinetics of the production and clearance of Aβ in patients with diabetes [13, 14]. Acute hyperglycemia induced by glucose clamps increased the levels of Aβ in the hippocampal interstitial fluid of APP/PS1dE9 transgenic mice [15]. Long-term hyperglycemia and hyperlipidemia on APP/PS1dE9 transgenic mice induced by high-fat diets are associated with elevated cortical levels of Aβ and overactivated astrocytes and microglia [16–18]. These studies suggest that chronic hyperglycemia, and elevated neuronal glucose levels are associated with neuroinflammation and cognitive deficits in APP/PS1dE9 transgenic mice.

Chronic hyperglycemia and insulin resistance elicited by a high-sucrose diet are also associated with the upregulation of GFAP, Iba1, and IL-1β in APP/PS1dE9 transgenic mice, suggesting that astrocytes and microglia are activated [19]. Furthermroe, Xuefu Zhuyu decoction and Astragalus membranaceus-Polysaccharides, two traditional Chinese medicine used to treat metabolic syndrome ameliorate hyperglycemia and insulin resistance also attenuate microglial and astrocytic overactivation in APP/PS1dE9 transgenic mice [20, 21]. Taken together, sustained hyperglycemia and elevated neuronal glucose levels exacerbate the accumulation of Aβ and neuroinflammation, which suggests that restoration of glycemic homeostasis may represent a possible strategy to attenuate the progression of AD [20, 22].

There are two major pathways of Aβ clearance. First, Aβ can be delivered across the blood brain barrier into the blood circulation and drain through the basement membranes of vessels into the lymphatic system [23]. Second, astrocytes and microglia can degrade Aβ via internalization or releasing degradation enzymes [24]. Insufficient clearance of soluble oAβ contributes to synaptic dysfunction and cognitive deficits in AD. Scavenger receptor A- and clathrin-dependent internalization of oAβ by primary microglia and lysosomal cathepsin mediate oAβ degradation [25, 26]. Lysosomal acidification is required for the degradation of fibrillar Aβ and oAβ by primary microglia [25, 27]. Primary astrocytes can also uptake Aβ via low-density lipoprotein receptor-mediated endocytosis, and internalized Aβ is transported to lysosomes [28]. Previous investigations of endothelial, muscle, liver, and pancreatic β cells show that hyperglycemia increases mitochondrial reactive oxygen species and results in mitochondrial dysfunction [29, 30]. Clathrin-mediated endocytosis is an ATP-dependent process that can be blocked by mitochondrial uncouplers [31]. Lysosomal acidification is also ATP-dependent [32]. Therefore, chronically dysfunctional mitochondria decrease acidification of lysosomes and clathrin-mediated endocytosis [33]. In summary, functional mitochondria are critical for the uptake and lysosomal degradation of Aβ.

Although *in-vitro* cell models have revealed the possible molecular regulatory mechanisms that mediate glial Aβ clearance, cell-based assays mostly employ either primary microglia or primary astrocyte cultures, and thus cannot replicate the close interactions between microglia and astrocytes in the neuronal microenvironment [34–36]. Moreover, exposure of primary microglia and astrocyte cultures to acute increased glucose levels may not recapitulate the prolonged hyperglycemic microenvironment in the brain of patients with diabetes [37–39].

Until now, the impact of chronic hyperglycemia on glial Aβ clearance was unclear. To address these issues, we conducted long-term elevated glucose treatments of mixed glial cultures to provide a better cell-based model to investigate the molecular mechanisms of glial oAβ clearance under hyperglycemic conditions. We assessed whether sustained hyperglycemia reduced oAβ clearance and explored the underlying mechanisms using a mixed glia model system cultured *in vitro* in media in containing normal glucose (NG, 5 mM) or high glucose (HG, 25 mM) for 16 days. The schematic diagram of our experimental design to investigate the effect of chronic hyperglycemia on mixed glia was present in S1 Fig. The impact of sustained HG on lysosomal acidification and the mitochondrial membrane potential of microglia and astrocytes in the mixed glia cultures were simultaneously investigated. Our findings provide evidence that sustained hyperglycemia impairs mitochondrial function and ATP production, which may contribute to lysosomal dysfunction, and in turn attenuate the degradation of internalized oAβ in astrocytes and microglia. Therefore, hyperglycemia-induced insufficient oAβ clearance may accelerate the accumulation of Aβ in the early stages of AD.

## Materials and methods

### Preparation of mixed glia cultures and primary microglia cultures

All animal handling procedures were approved by the Yang-Ming University Institutional Animal Care and Use Committee (IACUC No: 1061211r, 30 July, 2019). Mixed glia cultures were prepared from the cortices of neonatal Sprague Dawley rats at postnatal day 3, as described previously [40]. Briefly, rat pops were anesthetized on ice to for 10 min and the cortical tissue was separated from the meninges and dissociated and triturated in Dulbecco’s Modified Eagle Medium (DMEM) containing papain and DNase I (Worthington Biochemical Corp., Freehold, NJ, USA; Cat. LS003126, LS002139). Cells were directly seeded on coverslides coated with poly-*L*-ornithine (Sigma, St. Louis, MO, USA; Cat. 4957) and cultured in 24-well plates in DMEM containing 10% low-endotoxin fetal bovine serum (Gibco®, Grand Island, NY, USA; Cat. 16000) with either 25 mM glucose (high glucose, HG) or 5.5 mM glucose (normal glucose, NG). The seeding density was 8 × 10 ^4^ for NG mixed glia and 7 × 10^4^ for HG mixed glia. The cells were cultured for 16 days *in vitro* (DIV); the media were changed at 1 and 7 DIV. The NG and HG mixed glia cultures were subjected to the predesigned experiments at DIV16.

To prepare primary microglia cultures, mixed glia were cultured in NG and HG growth medium for 16 days in a T75 flask coated with poly-*L*-ornithine. Primary microglia were purified by gentle agitation with mixed glia and cultured on coverslides coated with poly-*L*-ornithine for 24 h before conducting the experiments [25].

### Preparation of oligomeric Aβ (oAβ)

Aβ_1–42_ peptide and FAM-labeled Aβ_1–42_ peptide were purchased from Biopeptide (Sunnyvale, CA, USA, Cat. 1-800-909-2494); oAβ was prepared as previously described [25, 41]. Briefly, 1 mg Aβ_1–42_ peptide or FAM-labeled Aβ_1–42_ peptide were dissolved in 1 mM hexafluoroisopropanol for 1 h. After air drying, the peptide film was dissolved in dimethyl sulfoxide (Sigma; Cat. D2650), then diluted in Ham’s F12 medium (PAN-Biotech, Aidenbach, Bavaria, Germany; Cat. P04-14559) to a final concentration of 100 μM. After incubation at 4°C for 24 h, the solutions were centrifuged to remove fibrillar and insoluble Aβ aggregates. The supernatant was designated oAβ. NG and HG mixed glia were incubated with 4 μM oAβ which was calculated from the initial amount (1 mg) of Aβ_1–42_ monomer applied in the oligomerization reaction.

### Measurement of IL-1β by ELISA

The levels of IL-1β in the culture media of the primary microglia and mixed glia were assessed using an enzyme-linked immunosorbent assay (ELISA) kit according to the manufacturer’s instructions (R&D Systems, Minneapolis, MN, USA; Cat. DY501). Plates were read at a wavelength of 450 nm using a TECAN Genios reader (TECAN, Durham, NC, USA) and Magellan version 7.0 software.

### RNA extraction and real-time PCR

Total RNA was isolated from NG and HG mixed glia using TRIzol reagent (Invitrogen, Carlsbad, CA, USA; Cat 15596018) and treated with DNase (Promega, Madison, WI, USA; Cat. M6101). RNA was reverse transcribed into cDNA using SuperScript III (Invitrogen; Cat. 18080–051). Real-time polymerase chain reaction (PCR) was performed using SYBR Green PCR Master Mix (Invitrogen; Cat. 4367659); the primer sets were: 5’-GCTGG AGCAAGACAAACATTC-3’ (forward) and 5’-CCCTACCCA CTCCTA CATCGT-3’ (reverse) for *GFAP*; 5’-AATG ACCTGTTCTT TGAGGCT GAC-3’ (forward) and 5’-CGAGATGCTGCTGTGAGA TTTGAAG-3’ (reverse) for *IL-1β*; 5’-TCCTACCCCAACTTCCAATGC TC -3’ (forward) and ’-TTGGATGGTC TTGGTCCTTAGCC-3’ (reverse) for *IL-6*; 5’-ATG GCCCAGACCCTCACACTCA GA-3’ (forward) and 5’-CTCCGCTTGGTGGTTTG CTACGAC-3’ for tumor necrosis factor (*TNF*) α; and 5’-CATTGCTGACAGGATGC AGAAGG-3’ (forward) and 5’-TGCTGGAAGGTG GACAGTGAGG-3’ for actin.

### Immunocytochemistry

To characterize the cellular morphology of the primary microglia and mixed glia cultures, the cells were fixed in methanol at 4°C for 10 min and incubated with primary antibodies against ionized calcium binding adaptor molecule 1 (Iba1; Wako, Osaka, Japan; Cat. 019–19741, 1:1000) or glial fibrillary acidic protein (GFAP; Sigma; Cat. G3893, 1:2000) overnight at 4°C. Afterward, the cells were incubated with secondary antibodies conjugated to AlexaFluor 488 or 594 (Invitrogen; Cat. A-21202, A-21206, A-21203 and A-21207, 1:500). The nuclei were stained with 1 μM 4’,6-diamidino-2-phenylindol (DAPI; Sigma; Cat. D9542) and the cells were mounted in Vitashield (Vector Laboratories, Burlingame, CA, USA; Cat. H-1000).

Images of five randomly selected fields of view were captured using a Zeiss confocal microscope (LSM780) with a 20×/0.8 objective or 40×/1.4 oil objective lens (frame size, 1024 × 1024; bit depth, 8). DAPI, AlexaFluor 488, and AlexaFluor 594 (Invitrogen; Cat. A-21202, A-21206, A-21203 and A-21207, 1:500) were exited using 405, 488, and 561 nm lasers, with XYZ sampling rates of 0.42 × 0.42 × 0.5 μm for the 20× objective lens and 0.21 × 0.21 × 1.0 μm for 40×/1.4 oil objective lens at a scan speed of 8. Microglial skeletal analysis was performed using FIJI ImageJ software. The form factor (circularity) of microglia is defined as 4πA/P^2^, where *P* is the perimeter of the cell and *A* is the area of the cells [42]. For cells with the same area, cells with a higher extent of ramification have a larger perimeter and thus a smaller form factor.

To assess steady-state oAβ uptake by astrocytes and microglia, mixed glia were incubated with FAM-labeled oAβ for 5 min or 1 h and then immunostained with anti-GFAP or anti-Iba1 antibodies. The nuclei were stained with 1 μM DAPI. Due to the higher steady-state level of oAβ puncta internalized by microglia compared to astrocytes, a higher detector gain was used when taking images of astrocytic oAβ internalization. Images of five randomly selected regions of the coverslips were captured using a Zeiss confocal microscope (LSM780) with a 40×/1.4 oil objective lens. The average fluorescence intensities of oAβ puncta in microglia and astrocytes of the mixed glia were quantified using MetaMorph software 7.1.

For the pulse-chase experiments, mixed glia were incubated with oAβ for 1 h and chased by rinsing away the oAβ-containing medium and replacing it with DMEM for indicated periods. Then, mixed glia were incubated with an anti-Aβ antibody (AB10; Millipore, Burlington, MA, USA; Cat. MAB5208; 1:2000) and anti-Iba1 or anti-GFAP antibodies were used for immunostaining to investigate the degradation kinetics of internalized oAβ in a cell-type specific manner. Images of five randomly selected regions of the coverslips were captured using a Zeiss confocal microscope (LSM780) with a 40×/1.4 oil objective lens. The average fluorescence intensities of oAβ puncta in microglia and astrocytes in the mixed glia were quantified using MetaMorph software 7.1.

To assess membrane binding and early endocytosis of oAβ, mixed glia were incubated with FAM-labeled oAβ at 4°C for 30 min to slow vesicle movement, then the cells were immunostained using an anti-GFAP antibody. Images were captured using a Zeiss confocal microscope (LSM780) with a 40×/1.4 oil objective lens. To assess colocalization of internalized oAβ puncta and lysosomes, mixed glia cultures were incubated with FAM-labeled oAβ for 1 h at 37°C, immunostained with an anti-lysosomal associated membrane protein 1 (LAMP1) antibody (Abcam, Cambridge, UK. Cat. ab24170, 1:1000). Images were captured using a Zeiss confocal microscope (LSM780) with a 63×/1.4 oil objective lens (frame size, 1024 × 1024; bit depth, 8) with XYZ sampling rates of 0.13 × 0.13× 0.5 μm at a scan speed of 8.

### *In-vivo* microglial morphology analysis of mice under diet-induced hyperglycemia

The morphology of microglia in WT mice and APP/PS1dE9 transgenic mice (Mutant Mouse Resource and Research Center stock; Cat. 034832) carrying human mutant amyloid precursor protein (APP) and presenilin 1 (PS1) were investigated under normoglycemic and hyperglycemic conditions by immunohistochemistry. All animal handling procedures were approved by the Yang-Ming University Institutional Animal Care and Use Committee (IACUC No: 1061211r, 30 July, 2019). Five weeks after the dietary switch from normal chow to a 60% high-fat diet (Research Diet, New Brunswick, NJ, USA; Cat. D12492) plus high-fructose containing drinking water or to a 35% high-sucrose diet (Research Diet; Cat. D12450B), the levels of fasting glucose were measured using a glucometer (Bioptik Technology, Taipei, Taiwan). Fifteen weeks after the dietary switch, 35-week-old mice were anesthetized with 2% ~ 3% isoflurane. After 5 to 10 minutes, pedal reflex was examined to ensure the depth of anesthesia. When mice lost the pedal reflex, mice were perfused with 50 ml saline with heparin, following with 25–30 ml 4% paraformaldehyde. The brain tissues were cryoprotected with sucrose solutions. Free-floating brain sections were immunostained with anti-Iba1 antibody (1:300, Abcam) followed by a biotinylated goat anti-rabbit secondary antibody. The sections were washed with Tris-buffered saline, incubated with ABC reagent. After a DAB colorimetric reaction, slices were mounted, and imaged using an Olympus BX51 microscope with a 40×/0.75 objective lens. The form factor, branch length, and the number of end points of microglia were quantified using FIJI ImageJ software [43]. Briefly, the images of brain sections were transformed to 8-bit format, the Unsharp Mask and Despeckle were applied to increase contrast and remove noise, then the images were skeletonized and analyzed using the plugin AnalyzeSkeleton (2D/3D). Branch length smaller than10 μm was not neglected during analyzing the average branch length as previously described [44].

### Western blotting analysis

To assess astrocyte activation, mitochondrial biogenesis and lysosome biogenesis after sustained NG and HG culture, mixed glia were lysed in Cell Lysis Buffer (Cell Signaling Technology, Beverly, MA, USA; Cat. 9803). After electrophoresis, the proteins were transferred onto polyvinylidene difluoride membranes (Millipore; Cat. IPVH00010) and the membranes were incubated overnight at 4°C with primary anti-GFAP (Dako, Tokyo, Japan, Cat. Z0334, 1:1,000), anti-glyceraldehyde 3-phosphate dehydrogenase (GAPDH; GeneTex, Irvine, CA, USA, Cat. GTX100118, 1:10,000), anti-LAMP1 (Abcam; Cat. ab24170, 1:1000), or anti-voltage-dependent anion-selective channel 1 (VDAC1; Millipore; Cat. MABN504; 1:1000) antibodies. After incubation with goat anti-rabbit (Sigma; Cat. A0545) or goat anti-mouse (Sigma; Cat. A9917) secondary antibodies, the immune complexes were detected using a chemiluminescence kit (Millipore; Cat. WBLUF0500) and the band intensities were quantified using analyzed by FUJIFILM LAS4000 luminescent image analyzer (Fujifilm Life Science, USA).

### Assessment of lysosomal acidification and hydrolysis activity

Lysosomal acidification was assessed using LysoSensor Green DND-189 (Invitrogen; Cat. L7535). Mixed glia were incubated with 1 μM LysoSensor at 37°C for 5 min at DIV 16. To assess the impact of oAβ treatment on lysosomal acidification, mixed glia were incubated with 4 μM oAβ for 1 h and stained with LysoSensor. The nuclei were stained with 1 μM DAPI. As fixation removes the florescence of LysoSensor, images of five randomly selected regions of the coverslips of live cells were imaged directly using a ZEISS Axioplan 2 microscope with a 40×/0.75 objective lens. The characteristic, distinct sizes of the nuclei of astrocytes and microglia were used to distinguish the glial types during quantification of the florescence intensity of astrocytes and microglia using MetaMorph software 7.1.

To directly compare the acidification of glial lysosomes in the transition from the basal level to oAβ treated circumstance with and without oAβ treatment, the total LysoSensor florescence intensity of five randomly selected images of NG and HG mixed glia were divided by the total cell number using MetaMorph software 7.1.

Lysosomal hydrolysis activity was assessed using DQ Red bovine serum albumin (DQ-BSA; Invitrogen; Cat. D-12051) as a cleavage substrate [45]. Mixed glia were incubated with 10 μg/mL DQ-BSA at 37°C for 8 h at DIV 16, and immunostained using anti-Iba1 or GFAP antibodies, individually. The nuclei were stained with 1 μM DAPI. After incubating with secondary antibody conjugated with AlexaFluor 488, images of five randomly selected regions of the coverslips were captured using a Zeiss confocal microscope (LSM780) with a 63×/1.4 oil objective lens. DAPI, AlexaFluor 488, and cleaved DQ-BSA were exited using 405-, 488-, and 561-nm lasers, with XYZ sampling rates of 0.13 × 0.13× 0.5 μm at a scan speed of 8. The fluorescence intensities of cleaved DQ-BSA of astrocytes and microglia were quantified using MetaMorph software 7.1.

To identify lysosomal activities of microglia and astrocytes in a cell type-specific manner, NG and HG mixed glia were incubated with anti-Iba1 and anti-GFAP antibodies after 8-h incubation of DQ-BSA. AlexaFluor 488 conjugated secondary antibody and AlexaFluor 633 conjugated secondary antibody were used to bind anti-GFAP antibody and anti-Iba1 antibody. The nuclei were stained using 1 μM DAPI. DAPI, AlexaFluor 488, AlexaFluor 633, and DQ-BSA were exited using 405-, 488-, 633-, and 561-nm lasers.

### Measurement of mitochondria membrane potential

Mitochondria membrane potential was assessed using JC-10 (Enzo Life Sciences, Farmingdale, NY, USA; Cat. 52305). Mixed glia were incubated with 10 μg/mL JC-10 at DIV16 for 30 min at 37°C, then the nuclei were stained with 1 μM DAPI. As fixation removes the florescence of JC-10, live cells were imaged directly using a ZEISS Axioplan 2 microscope with a 40×/0.75 objective lens. The characteristic, distinct sizes of the nuclei of astrocytes and microglia were used to distinguish the glial types during quantification of the florescence intensity of astrocytes and microglia. The ratio of aggregated to monomer JC-10 (590 nm/525 nm) was calculated using MetaMorph software 7.1 as previously described [46].

### Measurement of ATP

The levels of ATP in mixed glia were measured using the ATP Colorimetric/Fluorometric Assay Kit (BioVision, Milpitas, CA, USA; Cat. K808). Briefly, NG and HG mixed glia were lysed at DIV16 in 100 μL ATP assay buffer. The lysates were deproteinized by adding ice-cold perchloric acid (Biovision; Cat. K354-100), vortexed, placed on ice for 5 min, centrifuged and neutralized. The supernatants were mixed with Reaction Mix containing ATP probe and incubated at room temperature for 30 min. The absorbance (OD 570 nm) values were measured using a TECAN Genios reader and Magellan version 7.0 software.

### Statistical analysis

Statistical analysis was performed using GraphPad Prism (GraphPad, San Diego, CA, USA). All values are given as mean ± standard error of the mean. All experiments were performed more than three times. Comparisons of two groups were performed using unpaired Student t. One-way analysis of variance (ANOVA) was followed by Bonferroni post hoc analysis in the morphological analysis of microglia *in vivo* and the comparison of Lysosensor intensity of NG and HG mixed glia with and without oAβ.

## Results

### Ramified microglia in mixed glia cultures replicates the in-vivo morphology of microglia in areas without senile plaques in APP/PS1dE9 transgenic mice

The majority of cell-based studies employ primary astrocyte or microglia cultures, which cannot replicate the dynamic interactions between astrocytes and microglia *in vivo*. Furthermore, previous studies applied drastically elevated glucose concentrations to mimic hyperglycemia. Therefore, we established primary mixed glial cultures and isolated microglia cultures and cultured the cells in media containing normal (physiological) glucose (5.5 mM, NG) or high glucose (25 mM, HG). We compared the morphologies and activation status of the cells after 16 days *in vitro* (DIV).

**Fig 1:**
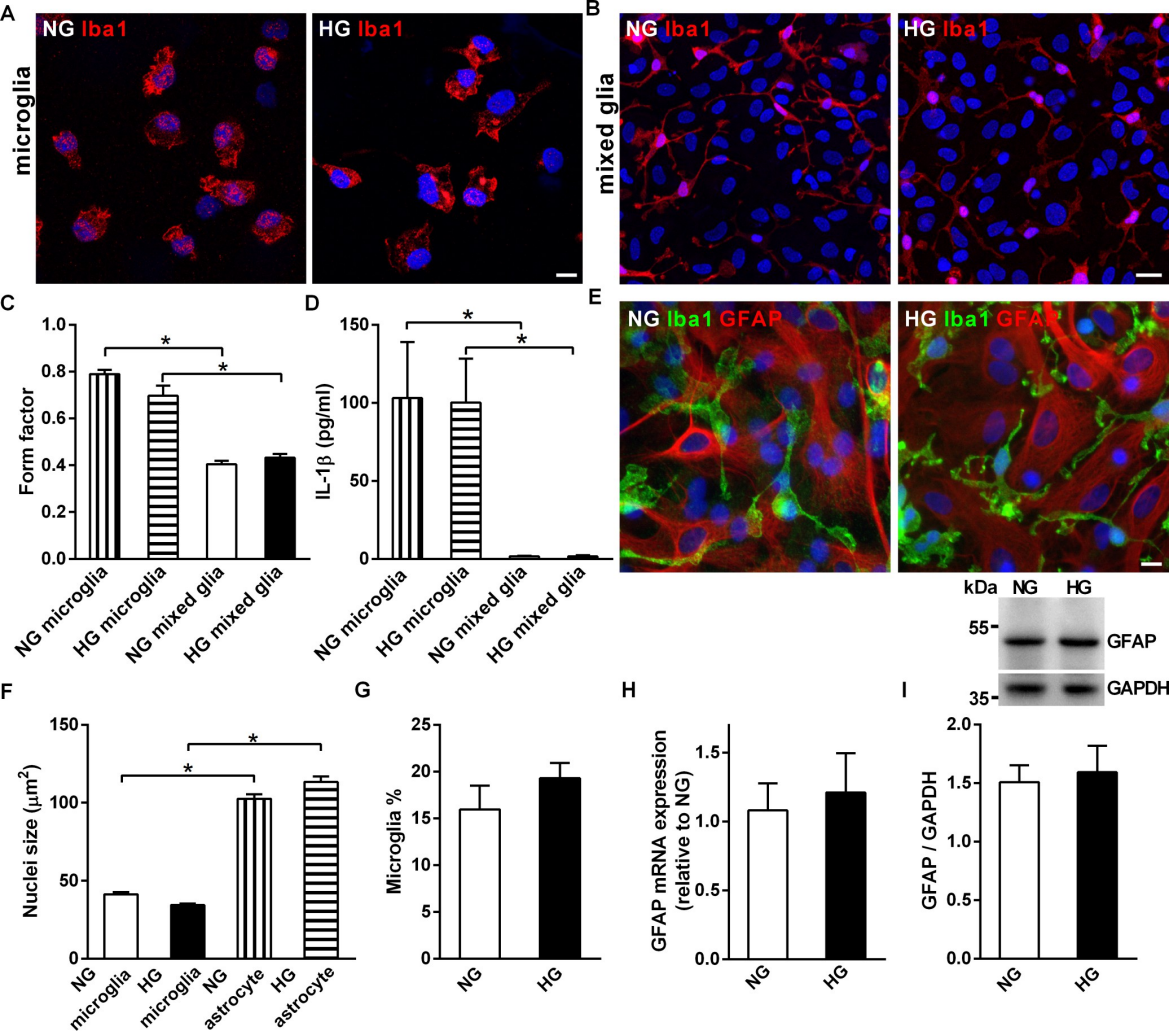
Comparison of the morphology of microglia in primary microglia cultures and mixed glia cultures. (A, B) Representative confocal images of microglia in primary microglia cultures and mixed glia cultures grown in media containing 5.5 mM (NG) or 25 mM (HG) glucose. Cells were immunostained with an anti-Iba1 antibody (red). (C) The form factor representing the ameboid feature of microglia in primary microglia and mixed glia cultures (D) Levels of secreted IL-1β in NG and HG primary microglia and mixed glia cultures. (E) Representative confocal images of NG and HG mixed glia cultures immunostained with anti-Iba1 antibody (green) and anti-GFAP (red) antibodies. (F) Nuclei size of microglia and astrocytes in NG and HG mixed glia cultures. (G) Percentages of microglia in NG and HG mixed glia cultures. (H, I) Real-time PCR and Western blot analysis of GFAP mRNA and protein levels in NG and HG mixed glia cultures. The molecular weights of the size markers are labeled on the left of the blot. The GFAP/GAPDH ratio was not significantly different between NG and HG mixed glia. Nuclei were stained using DAPI. NG, 5.5 mM glucose-containing media; HG, 25 mM glucose-containing media. Experiments were repeated at least three times. Data is expressed as mean ± SEM. Statistical differences between groups were determined by Unpaired Student’s t-test, and are labeled with *(p < 0.05). Scale bars, 10 μm (A, E) and 20 μm (B).

Isolated primary microglial cells exhibited an activated amoeboid morphology in both NG and HG culture media; however, the microglia in both NG and HG mixed glia cultures exhibited a ramified morphology (Fig 1A and 1B). The form factor is an index of cellular roundness; a higher value indicates cells with a rounder morphology [42]. Primary microglia had a significantly higher form factor than microglia in mixed glia (Fig 1C). Furthermore, the level of interleukin (IL)1-β protein was higher in NG and HG primary microglia than NG and HG mixed glial cultures (Fig 1D).

High magnification confocal images showed that the ramified microglia were in close contact with astrocytes in the mixed glia (Fig 1E). The nuclei sizes of astrocytes and microglia were significantly different, with astrocytes having larger nuclei (Fig 1F). The nuclei sizes of astrocytes and microglia were not affected by the concentration of glucose in the growth medium. In addition to cell-type specific immunostaining, the distinct nuclei sizes were also used to differentiate microglia from astrocytes in this study. The percentage of microglia (Fig 1G) and GFAP mRNA and protein expression (Fig 1H and 1I) in mixed glia were not significantly different under NG and HG conditions, suggesting that the ratio of microglia and astrocytes was comparable in NG and HG mixed glia.

The mRNA expression of IL-1β, but not IL-6 or TNF α increased in HG mixed glia compared to NG mixed glia, suggesting that HG mixed glia were mildly activated (S2 Fig). However, the mild activation induced by HG did not obviously alter the morphologies of the astrocytes or microglia in the mixed glial cultures (Fig 1B, 1C and 1E).

**Fig 2:**
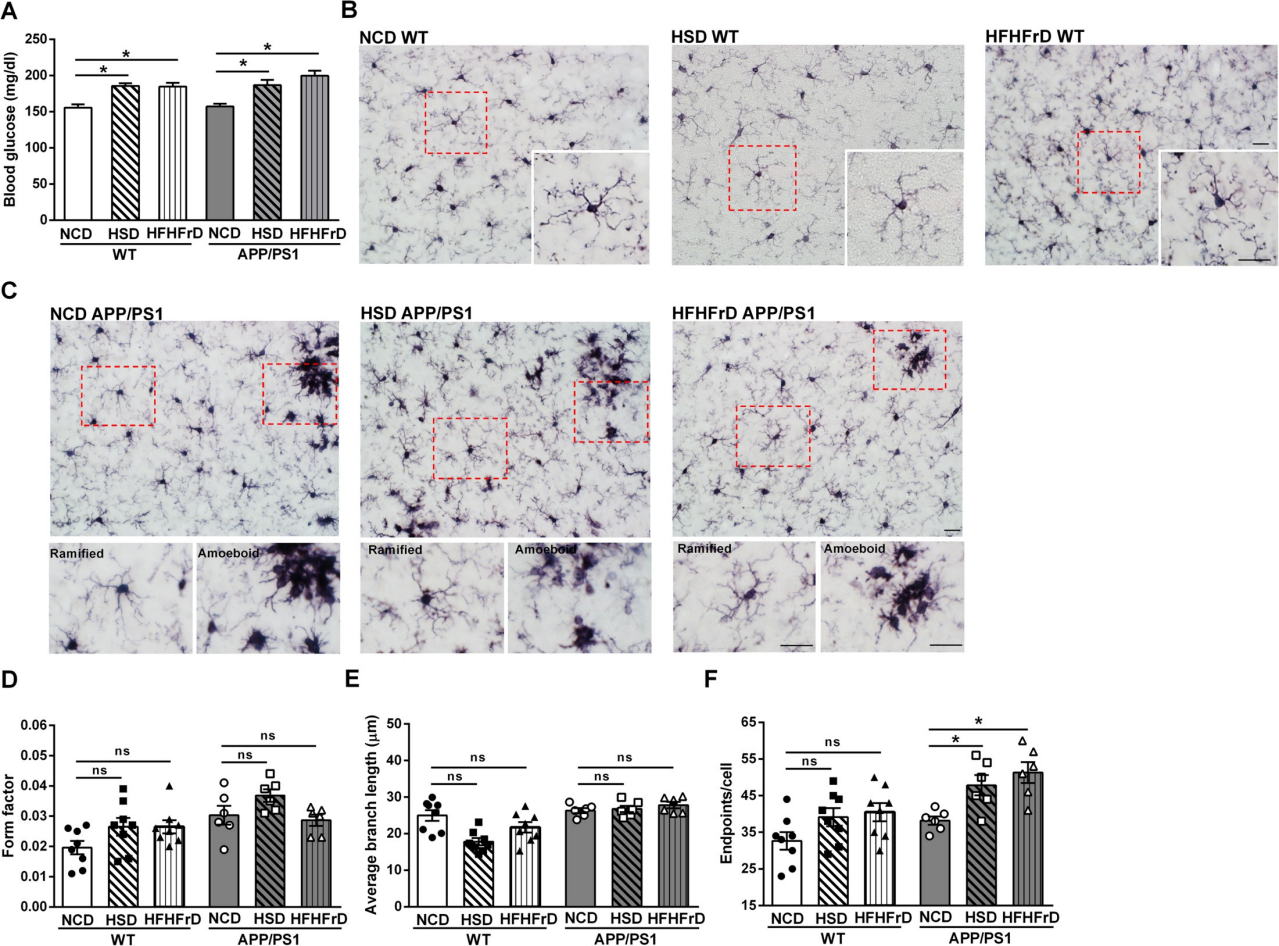
Ramified microglia in the brain parenchyma of wildtype mice and the brain parenchyma without senile plaques in APP/PS1dE9 transgenic mice under chronic hyperglycemia. (A) Fasting glucose levels of WT mice and APP/PS1dE9 transgenic mice on a normal chew diet (NCD) and after 5-week diet switch to a high-sucrose diet (HSD), or a high-fat diet plus fructose drinking water (HFHFrD). N = 10-15/group. (B) Representative images of the cortex of WT mice on NCD, HSD, and HFHFrD diets after immunostaining using anti-Iba1 antibody. The inserts show magnified images of the boxed areas. (C) Representative images of the cortex of APP/PS1dE9 transgenic mice on NCD, HSD, and HFHFrD (upper panels). The lower panels show magnified images of the boxed areas. (D) The form factor, (E) Average branch length, and (F) The number of endpoints of microglia in the cortex of WT mice and the cortex of APP/PS1dE9 transgenic mice in the area without senile plaques. Experiments were repeated at least three times. Eight microglia of WT mice and six microglia of APP/PS1dE9 transgenic mice were analyzed. Data is expressed as the mean ± SEM. Significant differences between groups were determined by one-way ANOVA followed by Bonferroni *post-hoc* tests, and are labeled using * (*p* <0.05). Scale bar, 20 μm. No significant difference between two groups is labeled ns.

Next, we examined whether the ramified microglia in mixed glia cultures replicate the *in vivo* morphology of microglia in WT mice and APP/PS1dE9 transgenic mice exposed to normoglycemia or hyperglycemia. Hyperglycemia was induced in WT mice and APP/PS1dE9 transgenic mice by exposure to a high-sucrose diet (HSD) or a high-fat diet with 30% fructose-containing drinking water (HFHFrD) for 5 weeks (Fig 2A). In WT mice, microglia exhibited a ramified morphology, regardless of the diet (normal chow [NCD], HSD or HFHFrD; Fig 2B, inserts were magnified images) at fifteen weeks after the dietary switch. As we expected, amoeboid microglia were observed in the foci of senile plaques in APP/PS1dE9 transgenic mice consuming NCD, HSD, and HFHFrD (Fig 2C with magnified images in the lower panels) at fifteen weeks after the dietary switch. Nevertheless, the microglia in brain regions without senile plaques remained ramified.

Microglial morphology was analyzed using FIJI ImageJ software [43]. The form factor of microglia in WT mice on HSD and HFHFrD was not different from that of NCD WT mice (Fig 2D). Similar observation was found in APP/PS1dE9 transgenic mice. The average branch lengths and number of end points of microglia in NCD WT mice were defined by ImageJ (S3 Fig). The average branch lengths and number of end points were not different between WT mice on NCD, HSD, or HFHFrD (Fig 2E and 2F).

The average branch lengths of microglia in the regions of the brain parenchyma without senile plaques were not different between APP/PS1dE9 transgenic mice fed NCD, HSD, or HFHFrD (Fig 2E). However, the number of end points was higher in APP/PS1dE9 transgenic mice fed the HSD or HFHFrD compared with APP/PS1dE9 transgenic mice fed the NCD (Fig 2F). Therefore, hyperglycemia did not induce morphological alterations to the microglia in either the WT mice and APP/PS1dE9 transgenic mice. Our data indicates that microglia in the mixed glia cultures are in a relative resting state, similar to the ramified morphology of microglia in the brain parenchyma of WT mice and APP/PS1dE9 transgenic mice under normoglycemia or hyperglycemia *in vivo*. Therefore, mixed glia cultures provide a better *in vitro* model to investigate the effects of sustained HG on glial oAβ clearance, which is critical to understand the pathogenesis of AD.

### Chronic high glucose conditions attenuate oAβ uptake by astrocytes and microglia in mixed glia cultures

Western blot analysis of oAβ and FAM-labeled oAβ species was performed after oligomerization (S4A Fig). No aggregates retained in the gel wells. Trimers, tetramers, and a cluster of high-molecular weight species ranging from 70 kDa to180 kDa of oAβ and FAM-labeled oAβ species were detected.

**Fig 3:**
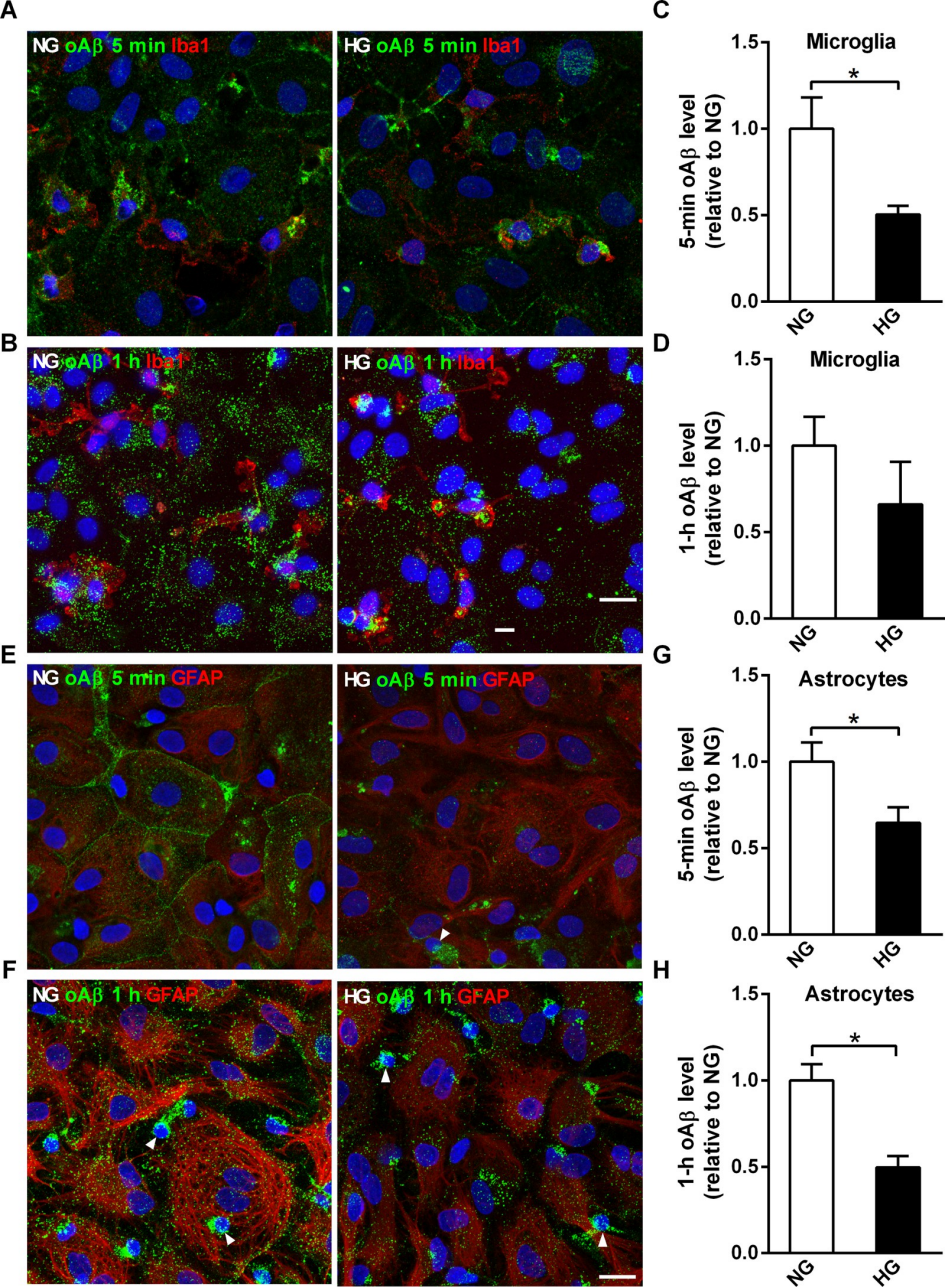
High glucose-containing media reduces the steady-state levels of internalized oAβ in microglia and astrocytes in mixed glia cultures. NG and HG mixed glia cultures were incubated with FAM-oAβ for 5 min or 1 h, then subjected to immunocytochemical analysis using anti-Iba1 (red) or anti-GFAP antibodies. (A, B) Representative confocal images of internalized FAM-oAβ puncta in microglia (red). (C, D) Quantification of the relative intensity of internalized oAβ puncta (green) in microglia (Iba1-positive, red) in NG and HG mixed glia. (E, F) Representative confocal images of internalized oAβ puncta in astrocytes (red). (G, H) Quantification of the relative internalization of oAβ puncta in astrocytes (GFAP-positive, red) in NG and HG mixed glia. Nuclei were stained with DAPI. Arrowheads indicate microglia, which have smaller nuclei compared to astrocytes. NG, 5.5 mM glucose-containing media; HG, 25 mM glucose-containing media. Experiments were repeated at least five times. Data is expressed as mean ± SEM. Statistical differences between groups were determined by Unpaired Student’s t-test, and are labeled with *(*p* < 0.05). Scale bar, 20 μm.

The impact of sustained high glucose conditions on the steady state levels of glial oAβ uptake was investigated by incubating mixed glia with FAM-labeled oAβ. Cross-sectional confocal images indicated that astrocytes in both NG and HG mixed glia can internalize oAβ (S4B Fig). NG and HG mixed glia were incubated with oAβ at 37°C to assess the steady state of intracellular oAβ puncta under physiological conditions. The steady-state level of oAβ puncta of microglia in NG mixed glia was higher after 5-min incubation compared to the microglia in HG mixed glia, but not after 1-h incubation (Fig 3A–3D). Due to the lower florescent signal of FAM-labeled oAβ uptake by astrocytes compared with microglia, a higher detector gain during confocal imaging was necessary to assess astrocytic oAβ internalization (Fig 3E and 3F). The steady-state levels of oAβ puncta in astrocytes in NG mixed glia was higher than astrocytes in HG mixed glia after both 5-min and 1-h incubations (Fig 3G and 3H).

Next, we examined whether the elevated steady-state level of internalized oAβ puncta in NG mixed glia was due to increased membrane binding and endocytosis events. NG and HG mixed glia were incubated with FAM-labeled oAβ at 4°C to slow down the membrane binding and early events in the endocytosis of oAβ puncta. Confocal imaging of FAM-labeled oAβ puncta on the plasma membrane and cytoplasm suggested more membrane binding and endocytosis of oAβ in NG mixed glia correlated with the higher steady-state level of oAβ puncta in NG mixed glia (S5 Fig).

### Chronic high glucose conditions attenuate oAβ degradation in mixed glia cultures

**Fig 4:**
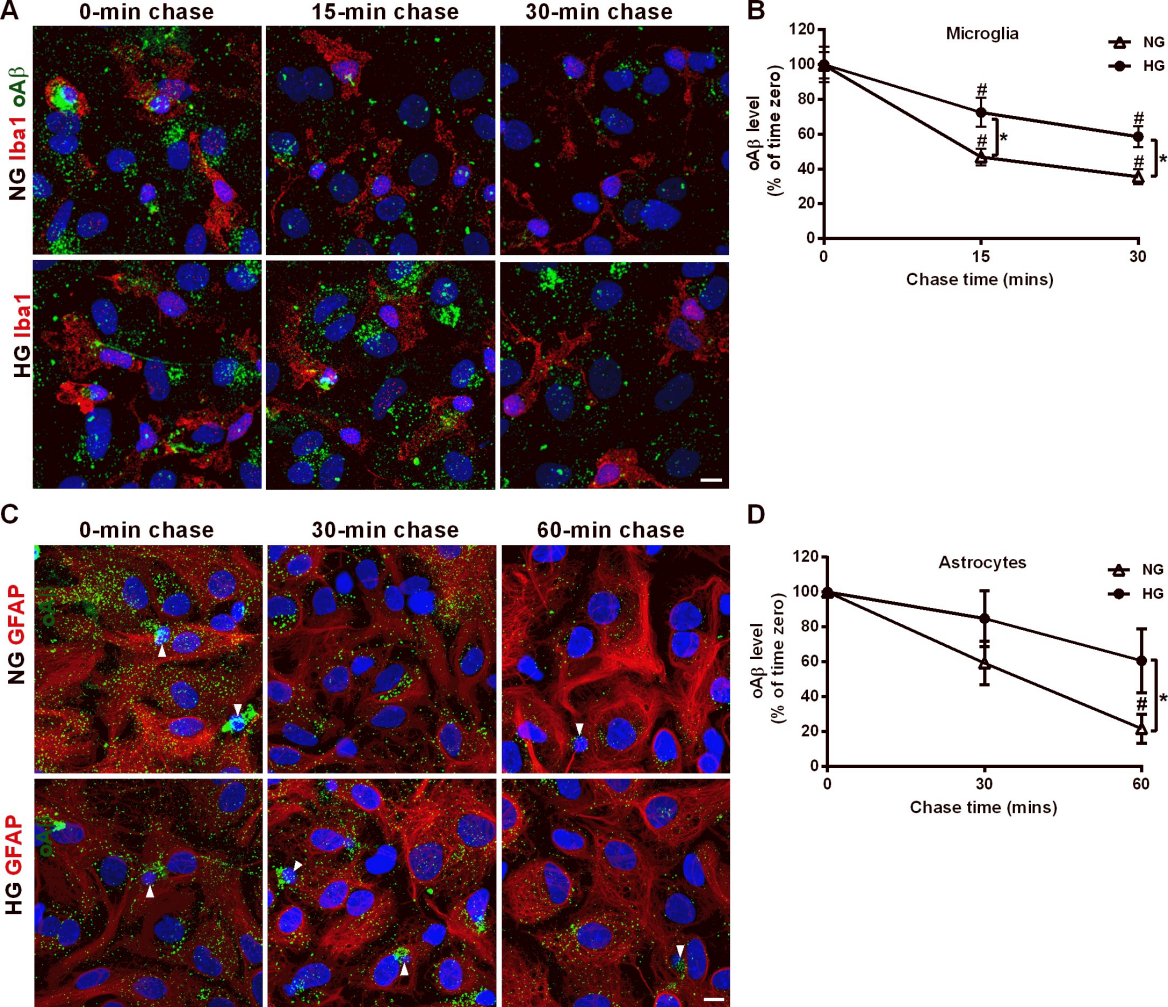
High glucose-containing media attenuates the degradation of internalized oAβ in mixed glia cultures. (A) NG and HG mixed glia were incubated with oAβ for 1 h, rinsed with DMEM and chased for 15 and 30 mins. Cells were immunostained using anti-Iba1 and anti-Aβ antibodies. Representative confocal images of microglia (red) and internalized oAβ puncta (green) after immunostaining. (B) Relative fluorescence intensity of oAβ puncta remaining in microglia after 15- and 30-min chase normalized to time zero. (C) NG and HG mixed glia were incubated with oAβ for 1 h, rinsed with DMEM and chased for 30 and 60 min. Immunocytochemical analysis was performed using anti-GFAP and anti-Aβ antibodies. Representative confocal images of astrocytes (red) and internalized oAβ puncta (green) after immunostaining. Arrowheads indicate microglia, which have smaller nuclei compared to astrocytes. (D) Relative fluorescence intensity of internalized oAβ puncta in astrocytes after 30- and 60-min chase normalized to time zero. Microglia (marked with arrowhead) were excluded during quantification of internalized oAβ in astrocytes. Nuclei were stained using DAPI. NG, 5.5 mM glucose-containing media; HG, 25 mM glucose-containing media. Data is expressed as mean ± SEM. Statistical differences between groups were determined by Unpaired Student’s t-test. ^#^ *P* < 0.05, chase timepoints vs. time zero; *P < 0.05, HG mixed glia vs. NG mixed glia. Scale bar, 10 μm.

Next, the degradation kinetics of internalized oAβ puncta in NG and HG mixed glia were investigated by the pulse and chase experiment. After 1-h incubation with oAβ, NG and HG mixed glia were chased for 15 and 30 min (Fig 4A). The fluorescence intensity of the oAβ puncta in microglia was significantly lower after 15- and 30-min chase than at time zero in both NG and HG mixed glia, suggesting that microglia in NG and HG mixed glia degraded internalized oAβ effectively (Fig 4B). Such reduction reaches plateau at 40% oAβ of time zero after 30-min chase in NG microglia in mixed glial cultures. Furthermore, higher levels of oAβ puncta were retained by microglia in the HG mixed glia than NG mixed glia at 15- and 30-min post-chase. It indicated that HG attenuated microglial oAβ degradation.

Due to the slower oAβ degradation kinetics in astrocytes, mixed glia were chased for 30 and 60 min to assess the retention of oAβ puncta in astrocytes after two chase periods (Fig 4C). The level of oAβ uncta in astrocytes in NG mixed glia at 30-min post-chase was not reduced compared with that at the time zero (Fig 4D). The level of oAβ puncta in astrocytes in NG mixed glia decreased significantly at 60-min post-chase compared with that at time zero, but not at 30-min post-chase. However, the levels of oAβ puncta retained by HG mixed glia were not statistically different to the levels at time zero even at 60-min post-chase. This finding suggests that HG slows down the kinetics of oAβ degradation in both microglia and astrocytes, and that the effects are more severe in astrocytes in mixed glia cultures.

### High glucose conditions reduce lysosomal acidification and hydrolysis activity in mixed glia cultures

**Fig 5:**
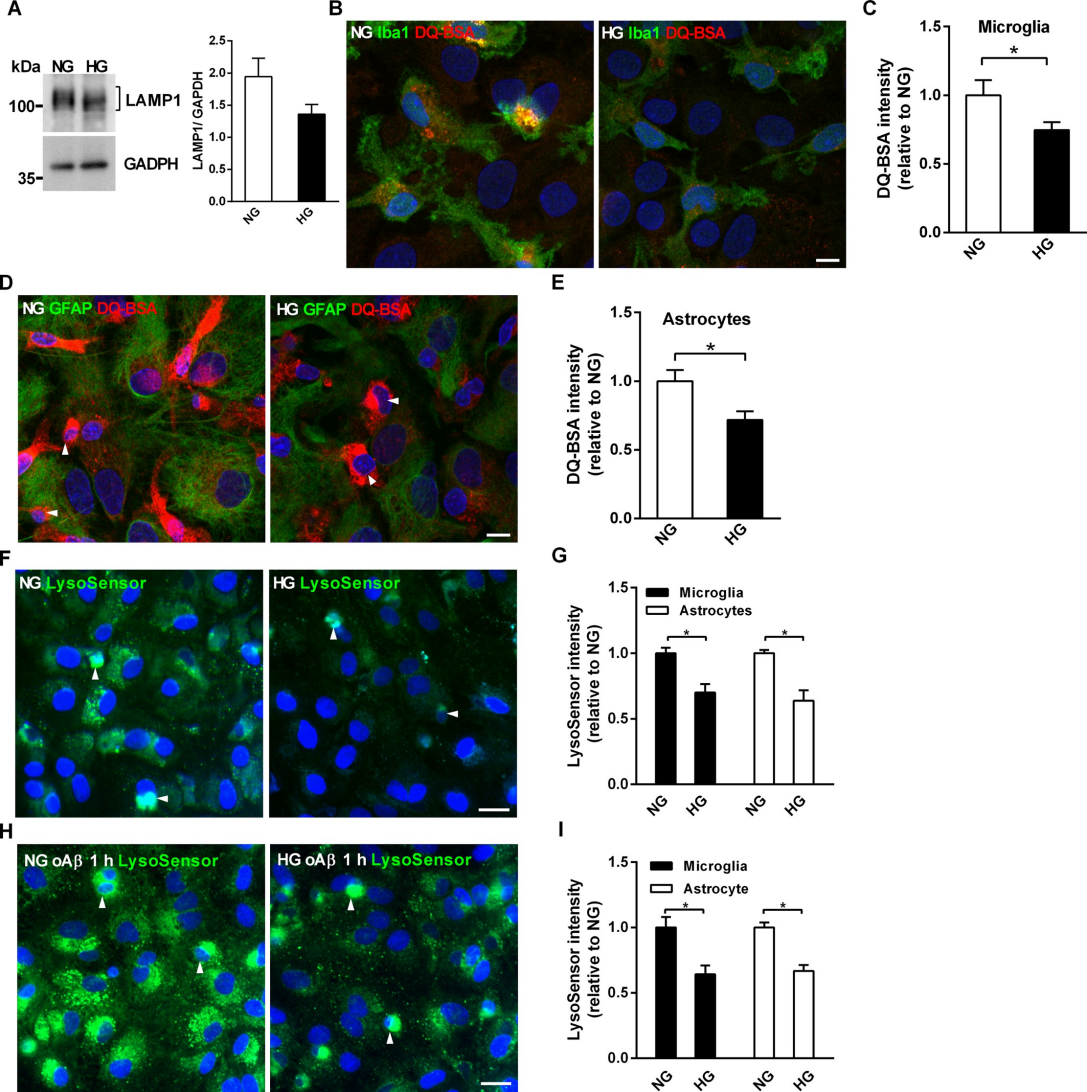
High glucose-containing media reduces lysosomal acidification and hydrolase activity in mixed glia cultures. (A) Western blot analysis of LAMP1 protein levels in NG and HG mixed glia cultures. The molecular weights of size markers are labeled on the left of the blot. (B) Representative confocal images of mixed glia incubated with DQ-BSA (red) and immunostained with anti-Iba1 (green). (C) Quantification of the relative fluorescence intensity of cleaved DQ-BSA in microglia. (D) Representative confocal images of mixed glia incubated with DQ-BSA (red) and immunostained with GFAP antibodies (green). (E) Quantification of the relative fluorescence intensity of cleaved DQ-BSA in astrocytes. (F) Representative fluorescent images of LysoSensor in live mixed glia at DIV16. (G) Relative LysoSensor fluorescence intensities in astrocytes and microglia in live NG and HG mixed glia at DIV16. (H) Representative LysoSensor fluorescence images of live mixed glia after 1-h oAβ incubation. (I) Quantification of the relative LysoSensor fluorescence intensities of astrocytes and microglia in live NG and HG mixed glia after 1-h oAβ incubation. Nuclei were stained with DAPI. Arrowheads indicate microglia, which have smaller nuclei compared to astrocytes. NG, 5.5 mM glucose-containing media; HG, 25 mM glucose-containing media. Experiments were repeated at least five times. Data is expressed as mean ± SEM. Statistical differences between groups were determined by Unpaired Student’s t-test, and are labeled with *(*p* < 0.05). Scale bars, 20 μm (B, H) and 10 μm (D, E).

We previously showed that lysosomal acidification is critical for oAβ degradation in primary microglia [25]. Similarly, internalized oAβ puncta highly co-localized with lysosomal-associated membrane protein 1 (LAMP1) in astrocytes in NG and HG mixed glia, suggesting that a large proportion of internalized oAβ are targeted to lysosomes (S6 Fig). Thus, we assessed whether the faster kinetics of oAβ degradation in NG mixed glia are related to altered lysosomal biogenesis. The levels of glycosylated LAMP1 were not significantly different in HG and NG mixed glia at DIV 16, suggesting that altered lysosomal biogenesis was not involved in the faster oAβ degradation kinetics in NG mixed glia (Fig 5A, *P* = 0.12).

Proteinase cleavage of DQ-BSA in lysosomes releases a florescent signal that can be used to visualize and quantify lysosomal protein degradation [45]. DQ-BSA was used to assess whether the reduced lysosomal acidification in HG mixed glia correlated with reduced acidic hydrolase activity in the lysosomes of HG mixed glia at DIV16. The confocal images of nuclei DAPI staining, DQ-BSA cleavage/DAPI and Iba1immunostaining/DAPI showed that dense florescence of cleaved DQ-BSA was observed in Iba1-positive microglia with smaller nuclei and cytoplasm (S7A Fig). The merged images showed that higher levels of DQ-BSA cleavage were observed in the microglia of NG mixed glia than HG mixed glia at DIV 16 (Fig 5B). Quantification of cleaved DQ-BSA showed the lysosomal hydrolysis activity was lower in both the microglia and astrocytes of HG mixed glia than those of NG mixed glia at DIV16 (Fig 5C).

Due to the weaker lysosomal activity of astrocytes compared to microglia, a higher detector gain during confocal imaging was necessary to detect DQ-BSA cleavage by astrocytes. The cleaved DQ-BSA was distributed evenly in GFAP-positive astrocytes with larger nuclei and cytoplasm (S7B Fig). The merged confocal images showed that higher levels of DQ-BSA cleavage were observed in astrocytes of NG mixed glia than HG mixed glia (Fig 5D). Similarly, the quantification of cleaved DQ-BSA showed lysosomal hydrolysis activity was lower in astrocytes of HG mixed glia than those of NG mixed glia (Fig 5E).

NG and HG mixed glia were subjected to the double immunostaining with anti-Iba1 and anti-GFAP antibodies to quantify lysosomal hydrolase activity in a cell-specific manner (S8A and S8B Fig). The cleaved DQ-BSA and nuclei size of Iba1-positive and GFAP-positive cells were quantified (S8C Fig). Consistent with the result shown in Fig 1B, the nuclei size of Iba1-positvie microglia was significantly smaller than that of GFAP-positive astrocytes (S8D Fig). The relative intensity of cleaved DQ-BSA in both Iba1-positive microglia and GFAP-positive astrocytes of NG mixed glia was higher than that of HG mixed glia (S8E Fig). It recapitulated the results shown in Fig 5B–5E Furthermore, the relative intensity of cleaved DQ-BSA in microglia was higher than that of astrocytes in HG mixed glia. Therefore, our data suggests that the hydrolysis activity of NG mixed glia was higher than HG mixed glia at the resting state.

Activation of lysosomal proteases requires acidification of lysosomes. Therefore, we investigated whether sustained HG affects lysosomal acidification. LysoSensor, a pH-dependent fluorescent indicator, was used to assess the pH levels of late endosomes and lysosomes. Since the fluorescence of LysoSensor is lost during fixation procedures, the distinct nuclei sizes of astrocytes and microglia were used to differentiate two glial cells while quantifying the intensity of LysoSensor in live NG and HG mixed glia. After culture for 16 days, the fluorescence intensity of LysoSensor was higher in NG mixed glia than HG mixed glia (Fig 5F). Quantification of the fluorescence intensity in live microglia and astrocytes, which were identified based on the size of their nuclei, showed that the lysosomes of both astrocytes and microglia were more acidified in NG mixed glia than HG mixed glia (Fig 5G). Next, we examined the effect of oAβ on the lysosomal acidification in NG and HG mixed glia. After 1 h of incubation with oAβ, the LysoSensor fluorescence intensity was higher in NG mixed glia than HG mixed glia (Fig 5H). Quantification of the LysoSensor fluorescence intensity revealed significantly higher lysosomal acidification in astrocytes and microglia of NG mixed glia than HG mixed glia after oAβ internalization (Fig 5I).

The LysoSensor fluorescence intensities of NG and HG mixed glia incubated with and without oAβ were compared directly to assess the lysosomal response to internalized oAβ puncta. 1-h oAβ treatment induced lysosomal acidification of NG mixed glia compared with that without oAβ treatment (S9A Fig). A similar enhancement in lysosomal acidification in response to 1-h oAβ incubation was observed in HG mixed glia (S9B Fig). Quantification of the LysoSensor fluorescence intensities showed that oAβ lysosomal targeting induced higher lysosomal acidification in NG mixed glia than in HG mixed glia (S9C Fig). In summary, HG attenuated the lysosomal acidification and hydrolysis activity of mixed glia with and without oAβ treatment.

### High glucose conditions reduce mitochondrial membrane potential in mixed glia cultures

**Fig 6:**
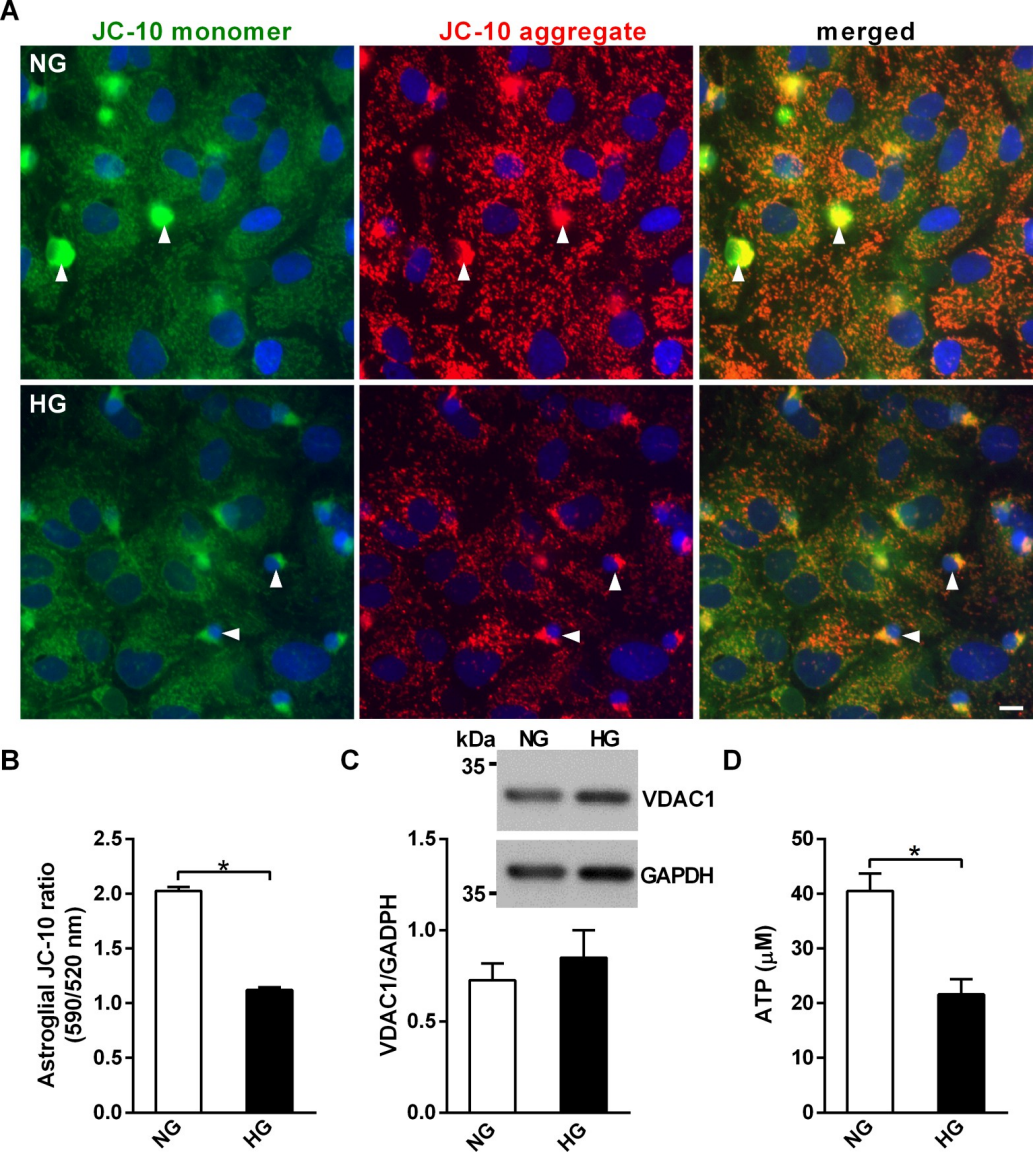
High glucose-containing media decreases mitochondrial membrane potential and ATP levels of in mixed glia cultures. NG and HG mixed glial cultures at DIV16 were stained with JC10 to assess mitochondrial membrane potential. (A) Representative fluorescent images of live mixed glia cultures stained with JC10. Arrowheads indicate microglia, which have smaller nuclei compared to astrocytes. (B) Mitochondrial membrane potential of astrocytes grown in NG and HG mixed glia. Microglia (marked with arrowhead) were excluded during quantification of the florescence of JC-10 in astrocytes. (C) Western blot analysis of the mitochondrial protein VDAC1 in NG and HG mixed glia. The molecular weights of size markers are labeled at the left of the blot. The VDAC1/GAPDH ratio was not significantly different between NG and HG mixed glia. (D) Cytosolic ATP levels in NG and HG mixed glia. NG, 5.5 mM glucose-containing media; HG, 25 mM glucose-containing media. Experiments were repeated at least three times. Data is expressed as mean ± SEM. Statistical differences between groups were determined by Unpaired Student’s t-test, and are labeled with *(*p* < 0.05). Scale bar, 10 μm.

Clathrin-dependent endocytosis and lysosomal acidification are both ATP-dependent [32]. Mitochondria actively modulates lysosomal activity during normal physical conditions and in neurodegeneration [33]. Therefore, we next examined whether HG-impaired mitochondrial function and ATP production may contribute to reduced oAβ clearance in HG mixed glia using JC-10, a probe for mitochondria membrane potential. Since the fluorescence of JC-10 is lost during fixation procedures, the distinct nuclei sizes of astrocytes and microglia were used to differentiate two glial cells while quantifying the intensity of JC-10 in live NG and HG mixed glia (Fig 6A). The ratio of the aggregated and monomer forms of JC-10 indicated that HG decreased the mitochondrial membrane potential of astrocytes (Fig 6B). The protein level of voltage-dependent anion channel 1 (VDAC1), an outer mitochondrial membrane protein, was not significantly different between NG and HG mixed glia, suggesting that mitochondria biogenesis was not involved in the higher florescence intensity of JC-10 in NG mixed glia (Fig 6C). As the microglial intensity of JC-10 monomers and aggregates were saturated in the NG mixed glia, the mitochondrial membrane potential of microglia could not be quantified. Consistently, the level of ATP was significantly higher in NG mixed glia than HG mixed glia (Fig 6D). Thus, these data suggest that HG reduces mitochondrial membrane potential and intracellular ATP, but not mitochondria mass, and these changes correlate with reduced lysosomal hydrolysis activity and glial oAβ degradation in mixed glia.

## Discussion

Although epidemiological studies indicate that diabetes is a risk factor for sporadic AD, the molecular mechanisms remain unclear. We and others have suggested that hyperglycemia and hyperlipidemia of prediabetes is associated with increased Aβ accumulation in APP/PS1dE9 transgenic mice [15–21]. Insufficient Aβ clearance correlates highly with the pathogenesis of AD, and glia play critical roles in Aβ clearance [47]. We hypothesized that chronic hyperglycemia reduces glial Aβ clearance and thus contributes to the pathogenesis of AD. Therefore, mixed glia cultures were established to investigate the impact of chronically elevated glucose concentrations on glial oAβ clearance. Overall, this study reveals that mitochondrial and lysosomal dysfunction induced by sustained hyperglycemia attenuate glial oAβ clearance.

Microglia in primary microglia cultures grown in NG and HG exhibited an activated, ameboid morphology and released high levels of IL-1β (Fig 1). In contrast, microglia coexisting with astrocytes in NG and HG mixed glia remained in an inactivated ramified form, and the release of IL-1β could not be detected. TGF-β released by astrocytes has been identified to remodeling microglial ramifications, suggesting that astrocytes actively modulate microglial functions [34]. Furthermore, we showed that ramified microglia were detected in WT mice under normoglycemia or hyperglycemic conditions. Consistently, Wanrooy et al. also showed that a high-fat diet does not induce gliosis or glial activation in WT mice [48]. The activated glial morphology was only observed at the foci of senile plaques *in vivo* in APP/PS1dE9 transgenic mice regardless normoglycemia or hyperglycemia (Fig 2). Rather than global activation, ramified microglia were detected in the remainder of the brain parenchyma of APP/PS1dE9 transgenic mice under normoglycemia or hyperglycemia. Therefore, previous investigations of the effects of hyperglycemia on Aβ clearance based on primary microglia or astrocyte cultures cannot replicate at least two *in vivo* aspects in patients with AD or AD transgenic mouse models. First of all, primary microglia and astrocytes are typically cultured individually *in vitro*. However, our mixed glia cultures replicate the active interactions between astrocytes and microglia [25, 49–51]. Secondly, the acute glucose levels used in most *in vitro* glial cultures are not physiologically relevant to chronic hyperglycemia in diabetes and patients with AD, as the glucose level in the brain parenchyma is relatively stable compared to that of the plasma [52]. Here, we avoided issues related drastic osmolarity shock by culturing mixed glia under sustained elevated glucose conditions without further disturbances.

This is the first study to simultaneously investigate the kinetics of oAβ uptake and degradation in ramified microglia and coexisting astrocytes under normoglycemia and sustained high glucose conditions. Microglia internalized oAβ more rapidly than astrocytes under both normoglycemia and sustained high glucose conditions. The steady-state level of oAβ puncta in microglia was lower under HG than NG at 5 min, but not after 1-h incubation (Fig 3). In contrast, the steady-state of oAβ puncta in astrocytes was lower in HG than NG after both 5-min and 1-h incubation. Pulse-chase experiments further suggested that the rate constant of oAβ degradation was lower in astrocytes than in microglia in NG (Fig 4B and 4D). Although the catalytic rate constant of oAβ degradation was lower in astrocytes than microglia, astrocytes can promote significant total internalization and Aβ degradation in mixed glial cell cultures because of their large cell size and high numbers. These results indicate that HG attenuates the degradation capabilities of glial cells.

After being internalized into glial cells, the majority of Aβ puncta are transported to early endosomes, late endosomes and lysosomes for degradation [25, 53]. The degradation of Aβ depends on acidification of late endosomes and lysosome complexes. The activity of hydrolase in microglia and astrocytes was decreased in HG compared to NG mixed glia. Low lysosomal acidification may result in low acidic hydrolase activity. The attenuation of lysosomal acidification by HG may contribute to lower degradation ability and increase the retention of internalized oAβ puncta in mixed glia. The protein level of LAMP1 was not different between HG and NG mixed glia, indicating that lysosomal biogenesis was not involved. Overall, our findings suggest that reduced lysosomal acidification attenuates the activity of hydrolase and decreases lysosomal Aβ degradation in HG mixed glia.

It has been shown that an acute high glucose challenge increases ATP production and mediates insulin secretion in isolated islet β-cells [54]. Consistently, elevated glucose level for 48 h enhances ATP production in insulinoma β-cells [55]. Increased ATP production and arrested proliferation of primary astrocytes are observed after being incubated in a medium containing 25 mM glucose for 48 h [39]. In addition to the cell culture systems, higher extracellular glucose also leads to increased ATP production and consequential neurotransmitter release in the nervous system under healthy condition [56]. However, reduced β-subunit of ATP synthase and ATP synthesis are observed in the liver and skeletal muscle of animal models and patients of hyperglycemia and diabetes, indicating that chronic hyperglycemia and insulin resistance reduce ATP production through downregulating the expression of β-subunit of mitochondrial ATP synthase [57–59]. Therefore, chronic hyperglycemia and an acute elevated glucose level may lead to distinct outcome on the ATP production.

Cell viability and intracellular vesicle trafficking are highly energy dependent. Glucose metabolism and energy production are the main functions of mitochondria. In this study, the mitochondria membrane potential was lower and the level of ATP level was reduced in HG mixed glia (Fig 5). Low level of ATP may result in lower proton pump activity, and thereby reduce lysosomal acidification [60]. Our data suggest that the low levels of ATP induced by HG may attenuate lysosomal acidification, and in turn lead to reduced oAβ internalization and degradation. Hyperglycemia may induce mitochondrial oxidative stress through 5’ AMP-activated protein kinase-independent pathways and inhibit transcription factor EB (TFEB), a critical transcription factor for lysosomal biogenesis and hydrolases [61]. Obese and diabetic conditions result in inactivation of TFEB and lysosomal protein degradation due to oxidative stress, suggesting hyperglycemia dysregulate the functions of mitochondria and lysosomes through inactivating TFEB [62, 63]. Three processes may explain how sustained high glucose conditions attenuate oAβ degradation by inhibiting the lysosomal activity of mixed glia. Firstly, a reduction in ATP production in a high glucose environment would inhibit glial lysosomal acidification. Secondly, inactivation of TFEB due to oxidative stress under high glucose conditions may attenuate lysosomal acidification. Thirdly, oxidative stress may directly destroy the integrity of the lysosomal membrane and disturb the lysosomal proton gradient.

Interestingly, 1-h oAβ treatment further enhanced lysosomal acidification in mixed glia (S9 Fig). We previously showed that the internalization of oAβ puncta involves the formation of dynamin-dependent and clathrin-coated vesicles [25, 26]. Since dynamin-activated ADP-ribosylation factor 6 enhances the assembly of proton pumps to increase lysosomal acidification, internalized oAβ clathrin-coated puncta may trigger glial lysosomal acidification by activating ADP-ribosylation factor 6 [64].

In conclusion, we demonstrate that mitochondrial and lysosomal dysfunction are associated with attenuated oAβ internalization and degradation in microglia and astrocytes in mixed glia after long-term exposure to high glucose conditions. Our data provide evidence that hyperglycemia potentiates oAβ accumulation which may lead to synaptic dysfunction. It may explain the high comorbidity of diabetes and AD. Furthermore, our study suggests that better long-term blood sugar control among patients with diabetes mellitus may effectively improve glial Aβ clearance and ameliorate the AD progression.

## Supporting information

S1 Fig The experimental schematic diagram. Mixed glia were cultured directly in 5.5 mM glucose-containing media (NG) and 25 mM glucose-containing media (HG) from day one *in vitro* (DIV 1). After 16 days *in vitro* (DIV 16), the basal levels of lysosomal hydrolytic activity and acidification of NG and HG mixed glia were compared. The mitochondrial membrane potential and ATP levels were measured. Next, NG and HG mixed glia were incubated with oAβ and the steady-state levels of internalized oAβ and the kinetic of oAβ degradation were quantified. The lysosomal response of internalized oAβ puncta was quantified using LysoSensor.
(TIF)

S2 Fig IL-1β mRNA expression is elevated in HG mixed glia. The mRNA levels of IL-1β, IL-6, and TNF α in NG and HG mixed glia cultures were quantified by real-time RT PCR. NG, 5.5 mM glucose-containing media; HG, 25 mM glucose-containing media. Data is expressed as mean ± SEM. Statistical differences between groups were determined by Unpaired Student’s t-test, and are labeled with *(*p* < 0.05).
(TIF)

S3 Fig Analysis of *in-vivo* microglial morphology. (A) Magnified image of microglia in the cortex of WT mice on a normal chew diet (NCD) after immunostaining using an anti-Iba I antibody. (B) The image of microglia was skeletonized and subjected to the morphological analysis using ImageJ. Branches longer than 10 μm and endpoints of microglia were labeled in green and blue and numbered.
(TIF)

S4 Fig Confocal images of oAβ internalization by astrocytes in NG and HG mixed glia cultures. (A) Western blot analysis of oAβ and FAM-oAβ after the oligomerization procedure. (B) NG and HG mixed glia cultures were incubated with FAM-oAβ for 1 h, then immunostained using an anti-GFAP antibody. Nuclei were stained using DAPI (blue). Representative confocal images of orthogonal projections of *z*-stacks containing six images acquired at intervals of 1 μm. NG, 5.5 mM glucose-containing media; HG, 25 mM glucose-containing media. Scale bar, 10 μm.
(TIF)

S5 Fig High glucose-containing media reduces the membrane binding and early endocytic events of oAβ in mixed glia cultures. NG and HG mixed glia were incubated with FAM-labeled oAβ at 4°C for 30 min. Immunocytochemical analysis was performed using an anti-GFAP antibody. Representative confocal images of astrocytes (red, upper panel), internalized oAβ puncta (green, middle panel), and merged images (lower panel). Nuclei were stained using DAPI (blue). NG, 5.5 mM glucose-containing media; HG, 25 mM glucose-containing media. Microglia are marked with arrowheads. Scale bar, 10 μm.
(TIF)

S6 Fig Internalized oAβ puncta colocalize with lysosomes in NG and HG mixed glia cultures. NG and HG mixed glia cultures were incubated with FAM-oAβ, then immunostained using an anti-LAMP1 antibody. Representative confocal images of lysosomes (red, upper panel), internalized oAβ puncta (green, middle panel), and merged images (lower panel). Nuclei were stained using DAPI (blue). NG, 5.5 mM glucose-containing media; HG, 25 mM glucose-containing media. Experiments were repeated at least three times. Scale bar, 10 μm.
(TIF)

S7 Fig The florescence of cleaved DQ-BSA is densely distributed in microglia with smaller nuclei and cytoplasm compared with astrocytes. Mixed glia were incubated with DQ-BSA and immunostained with anti-Iba1 or anti-GFAP antibodies. Nuclei were stained using DAPI. (A) Representative confocal images of nuclei of mixed glia (blue, left panel). Merged image of DAPI and immunostaining with anti-Iba1 (green) antibody (middle panel). Merged image of DAPI and DQ-BSA (red, right panel). (B) Representative confocal images of nuclei of mixed glia (blue, left panel). Merged image of DAPI and GFAP immunostaining (green, middle panel). Merged image of DAPI and DQ-BSA (red, right panel). Arrowheads indicate microglia, which have smaller nuclei compared to astrocytes. NG, 5.5 mM glucose-containing media; HG, 25 mM glucose-containing media. Scale bar, 50 μm.
(TIF)

S8 Fig Lysosomal hydrolase activities of astrocytes and microglia in mixed glia are quantified in a cell type-specific manner. (A, B) Representative confocal images of cleaved DQ-BSA (red) of NG and HG mixed glia cultures simultaneously immunostained with anti-Iba1 (white) and anti-GFAP (green) antibodies. Nuclei were stained using DAPI (blue). (C) The DQ-BSA cleavage activity and nuclei size of Iba I-positive microglia and GFAP-positive astrocytes in NG and HG mixed glia cultures (n = 16). (D) The nuclei size of Iba I-positive microglia and GFAP-positive astrocytes in NG and HG mixed glia cultures. (E) The DQ-BSA cleavage activity of Iba I-positive microglia and GFAP-positive astrocytes in NG and HG mixed glia cultures. Nuclei were stained using DAPI (blue). Arrowheads indicate microglia, which have smaller nuclei compared to astrocytes. NG, 5.5 mM glucose-containing media; HG, 25 mM glucose-containing media. Scale bar, 25 μm. Experiments were repeated at least three times. Data is expressed as the mean ± SEM. Statistical differences between groups were determined by Unpaired Student’s t-test, and are labeled with *(*p* < 0.05).
(TIF)

S9 Fig oAβ enhances lysosomal acidification in NG and HG mixed glia cultures. (A, B) Representative LysoSensor fluorescent images of live NG mixed glia and HG mixed glia at DIV16 incubated without treatment (left panel) and with oAβ for 1 h (right panel). (C) Quantification of the relative LysoSensor fluorescence intensity in NG and HG mixed glia incubated with and without oAβ. Nuclei were stained using DAPI (blue). NG, 5.5 mM glucose-containing media; HG, 25 mM glucose-containing media. Scale bar, 20 μm. Experiments were repeated at least three times. Data is expressed as the mean ± SEM. Significant differences between groups were determined by one-way ANOVA followed by Bonferroni *post-hoc* tests, and are labeled using * (*p* <0.05).
(TIF)

S10 Fig Raw images of GFAP Western blot. (TIF)

S11 Fig Raw images of oAβ Western blot. (TIF)

S12 Fig Raw images of LAMP1 Western blot. (TIF)

S13 Fig Raw images of VDAC1 Western blot. (TIF)
