# Supplementary figures and images for: Reduced mitochondria membrane potential and lysosomal acidification are associated with decreased oligomeric Aβ degradation induced by hyperglycemia: A study of mixed glia cultures
Source: PLoS One. 2022 Jan 24;17(1):e0260966. doi: 10.1371/journal.pone.0260966 (PMC8786178; doi:10.1371/journal.pone.0260966)

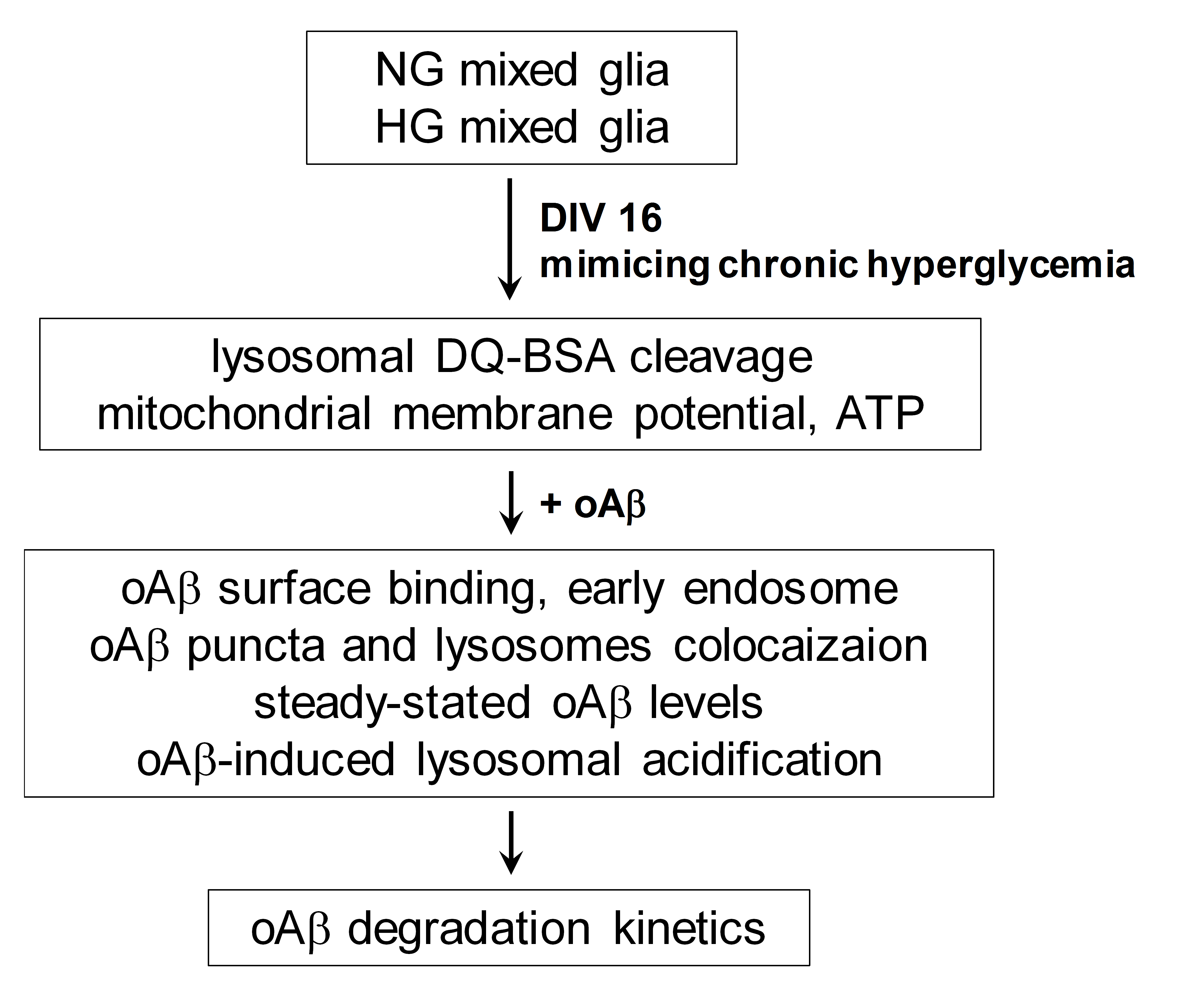

Supplement: S1 Fig — Mixed glia were cultured directly in 5.5 mM glucose-containing media (NG) and 25 mM glucose-containing media (HG) from day one in vitro (DIV 1). After 16 days in vitro (DIV 16), the basal levels of lysosomal hydrolytic activity and acidification of NG and HG mixed glia were compared. The mitochondrial membrane potential and ATP levels were measured. Next, NG and HG mixed glia were incubated with oAβ and the steady-state levels of internalized oAβ and the kinetic of oAβ degradation were quantified. The lysosomal response of internalized oAβ puncta was quantified using LysoSensor. (TIF) [file pone.0260966.s001.tif]

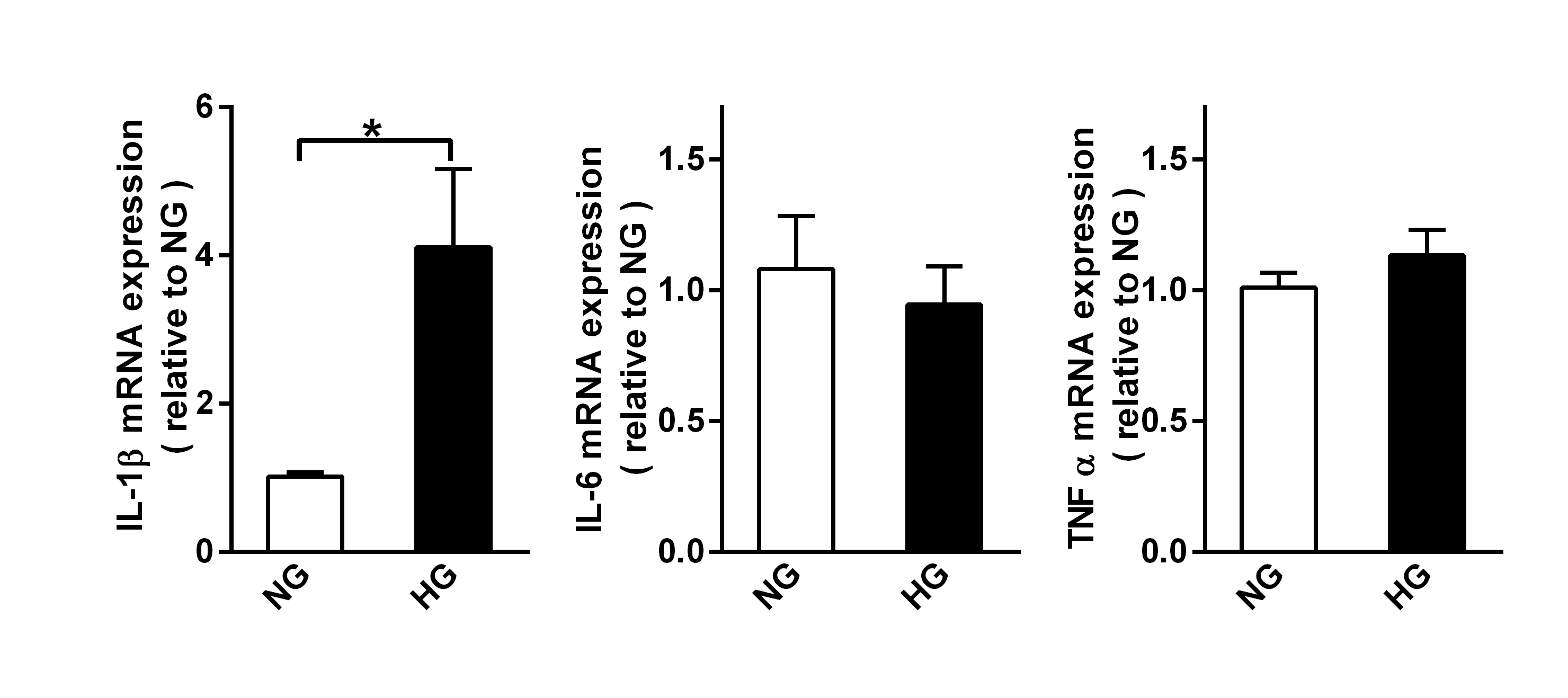

Supplement: S2 Fig — The mRNA levels of IL-1β, IL-6, and TNF α in NG and HG mixed glia cultures were quantified by real-time RT PCR. NG, 5.5 mM glucose-containing media; HG, 25 mM glucose-containing media. Data is expressed as mean ± SEM. Statistical differences between groups were determined by Unpaired Student’s t-test, and are labeled with *(p < 0.05). (TIF) [file pone.0260966.s002.tif]

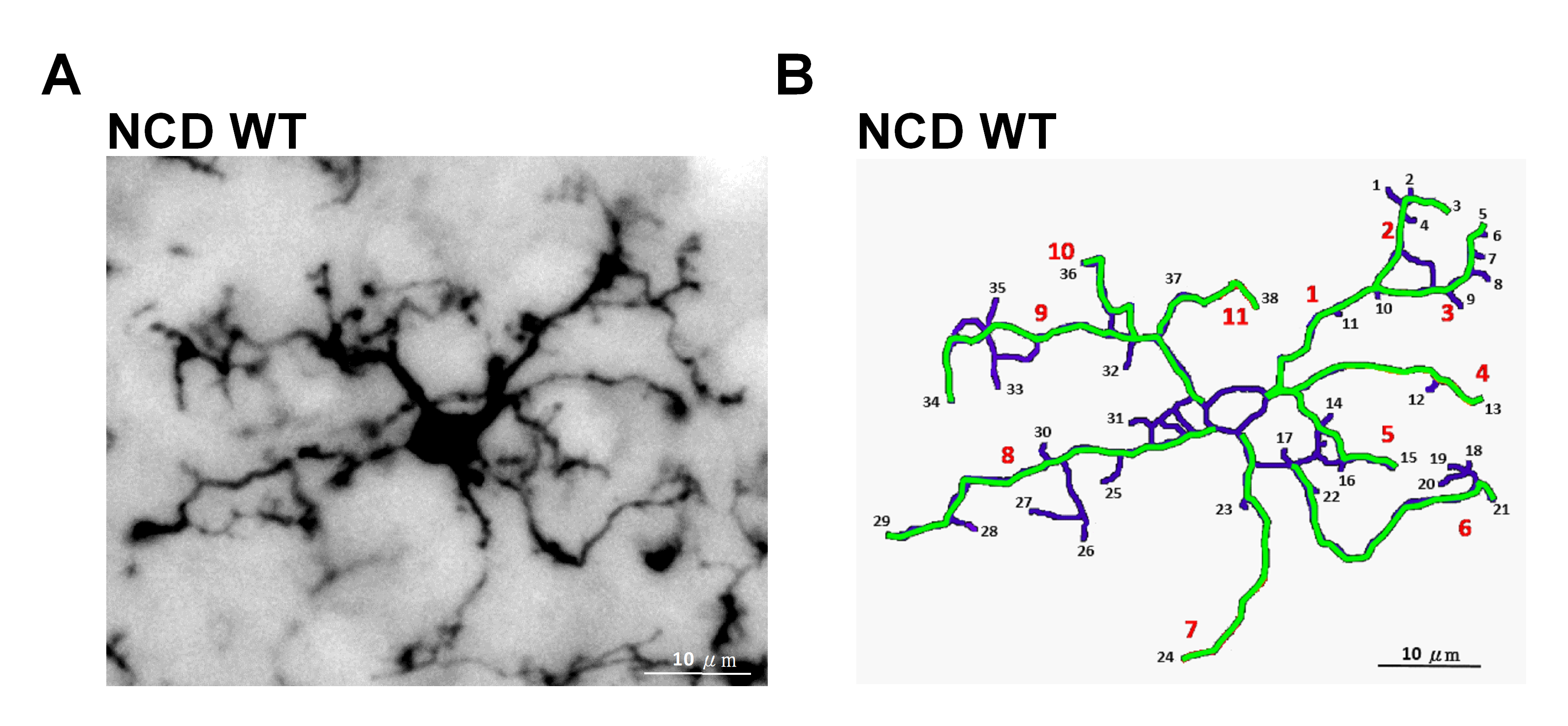

Supplement: S3 Fig — (A) Magnified image of microglia in the cortex of WT mice on a normal chew diet (NCD) after immunostaining using an anti-Iba I antibody. (B) The image of microglia was skeletonized and subjected to the morphological analysis using ImageJ. Branches longer than 10 μm and endpoints of microglia were labeled in green and blue and numbered. (TIF) [file pone.0260966.s003.tif]

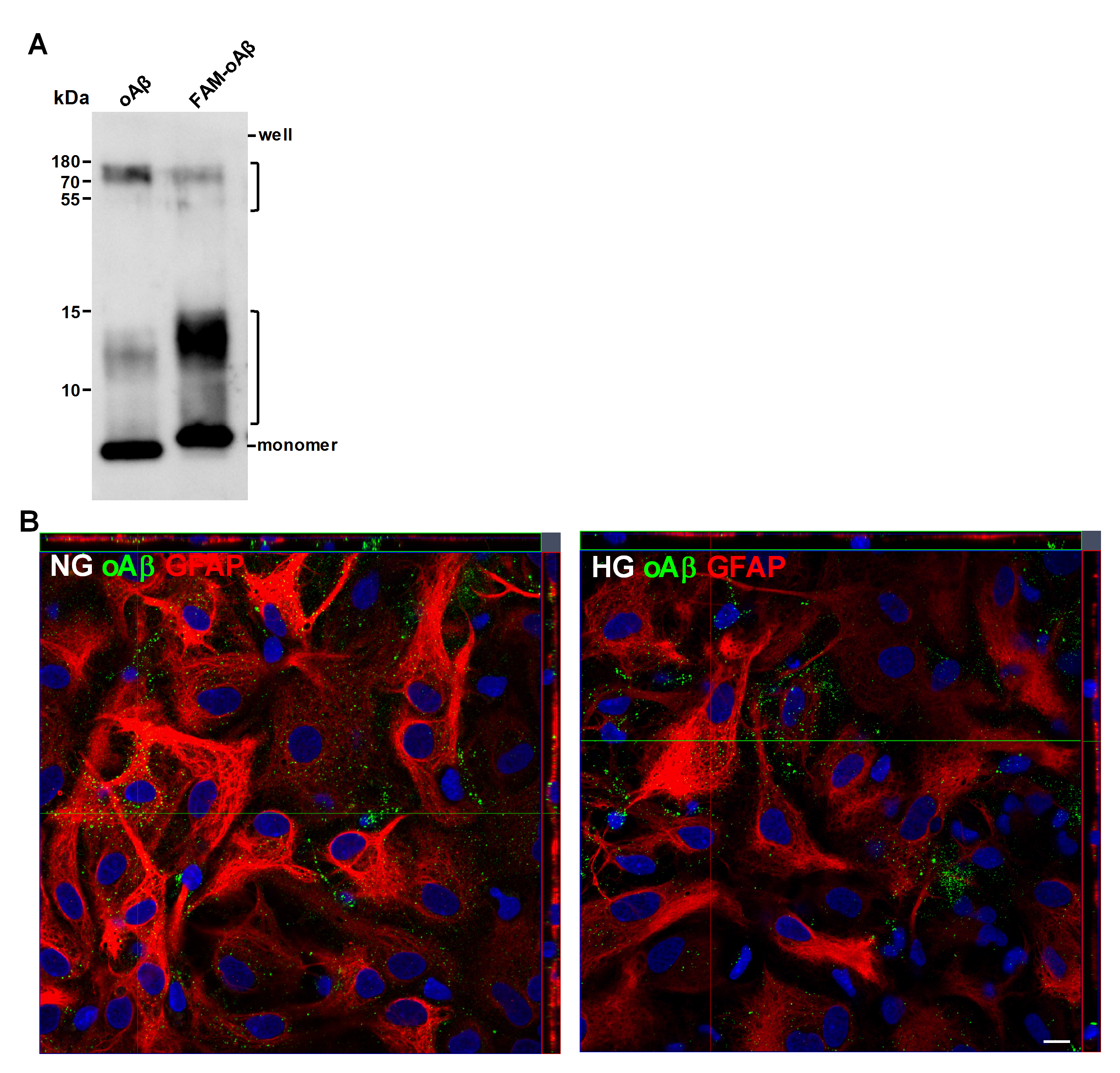

Supplement: S4 Fig — (A) Western blot analysis of oAβ and FAM-oAβ after the oligomerization procedure. (B) NG and HG mixed glia cultures were incubated with FAM-oAβ for 1 h, then immunostained using an anti-GFAP antibody. Nuclei were stained using DAPI (blue). Representative confocal images of orthogonal projections of z-stacks containing six images acquired at intervals of 1 μm. NG, 5.5 mM glucose-containing media; HG, 25 mM glucose-containing media. Scale bar, 10 μm. (TIF) [file pone.0260966.s004.tif]

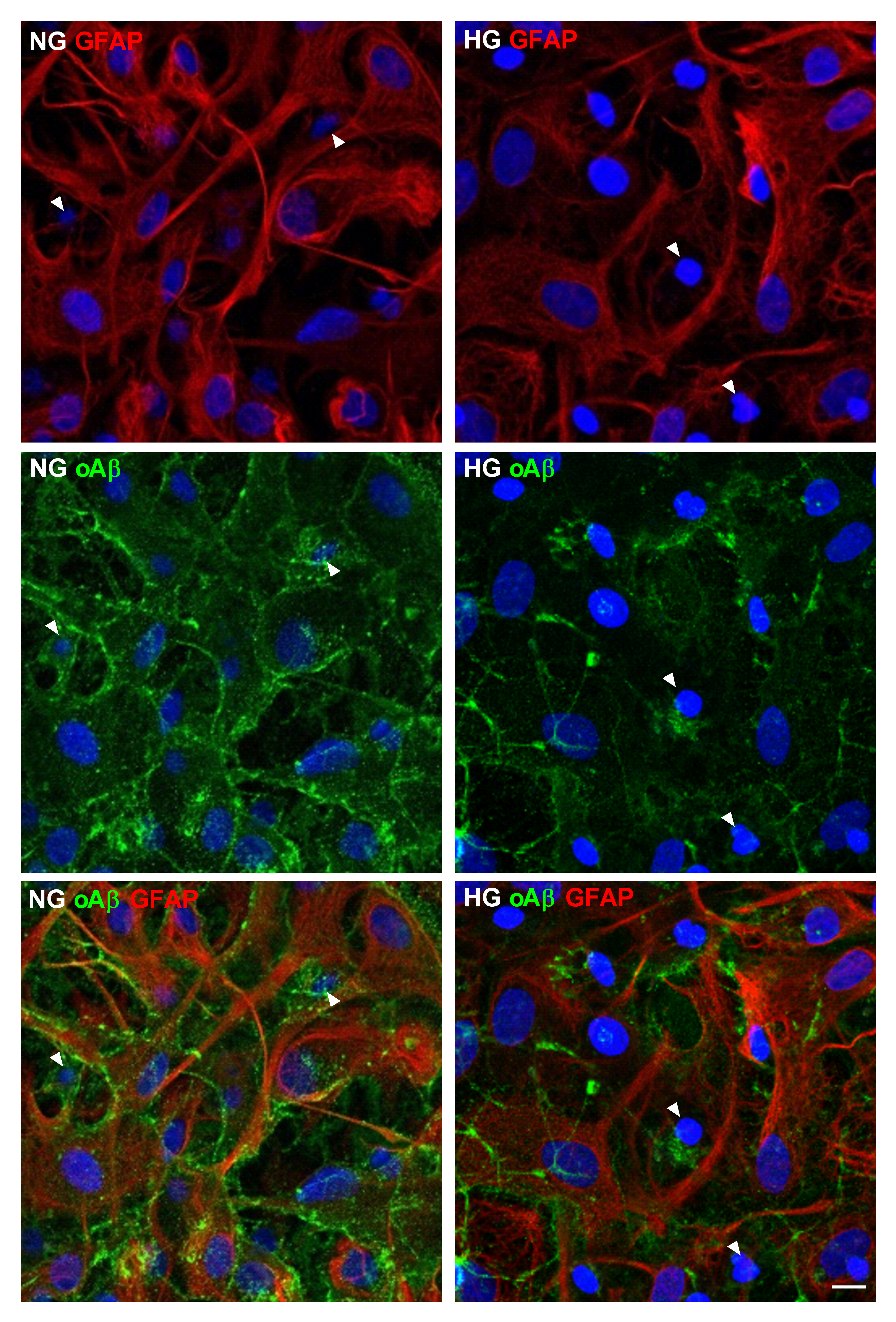

Supplement: S5 Fig — NG and HG mixed glia were incubated with FAM-labeled oAβ at 4°C for 30 min. Immunocytochemical analysis was performed using an anti-GFAP antibody. Representative confocal images of astrocytes (red, upper panel), internalized oAβ puncta (green, middle panel), and merged images (lower panel). Nuclei were stained using DAPI (blue). NG, 5.5 mM glucose-containing media; HG, 25 mM glucose-containing media. Microglia are marked with arrowheads. Scale bar, 10 μm. (TIF) [file pone.0260966.s005.tif]

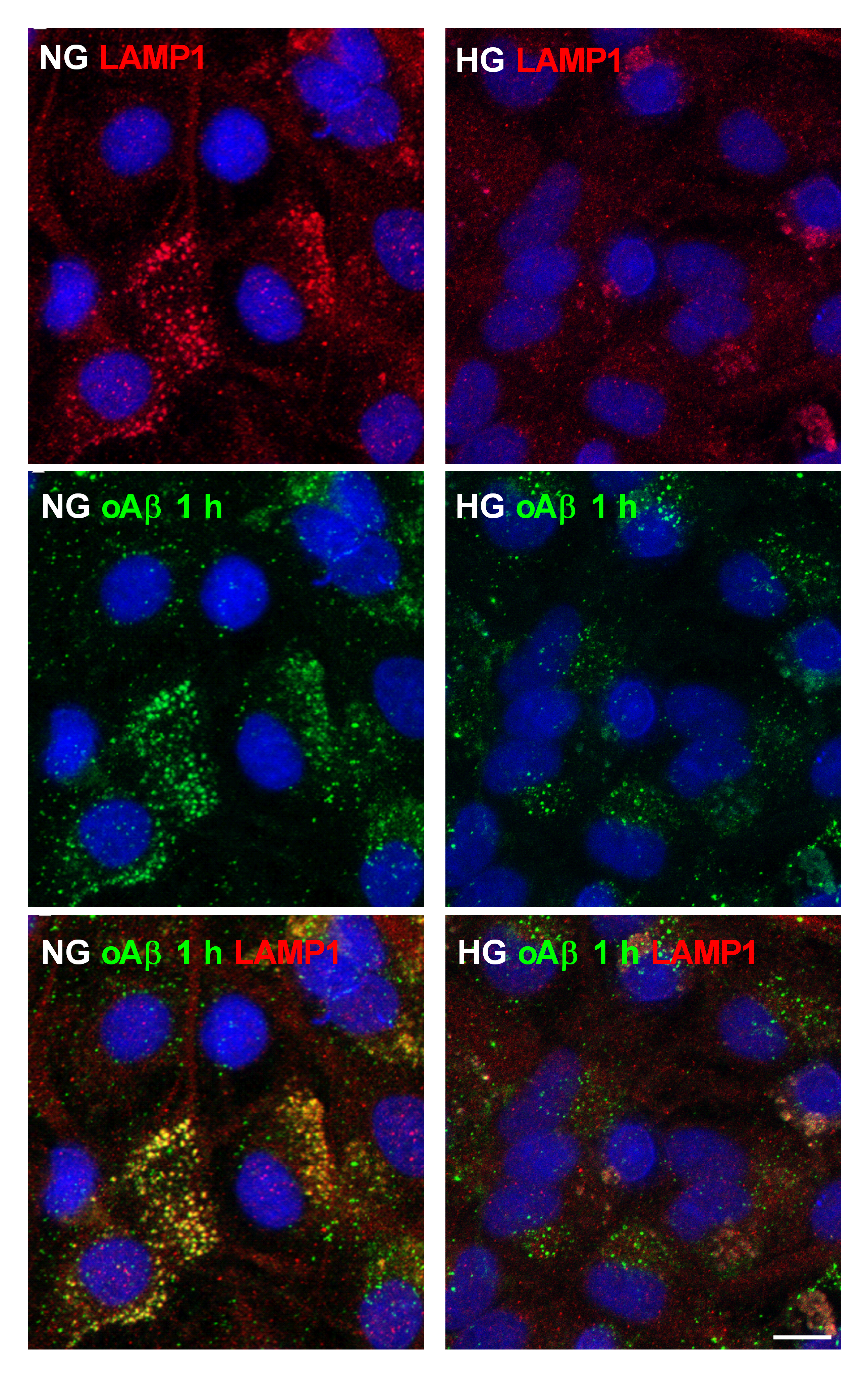

Supplement: S6 Fig — NG and HG mixed glia cultures were incubated with FAM-oAβ, then immunostained using an anti-LAMP1 antibody. Representative confocal images of lysosomes (red, upper panel), internalized oAβ puncta (green, middle panel), and merged images (lower panel). Nuclei were stained using DAPI (blue). NG, 5.5 mM glucose-containing media; HG, 25 mM glucose-containing media. Experiments were repeated at least three times. Scale bar, 10 μm. (TIF) [file pone.0260966.s006.tif]

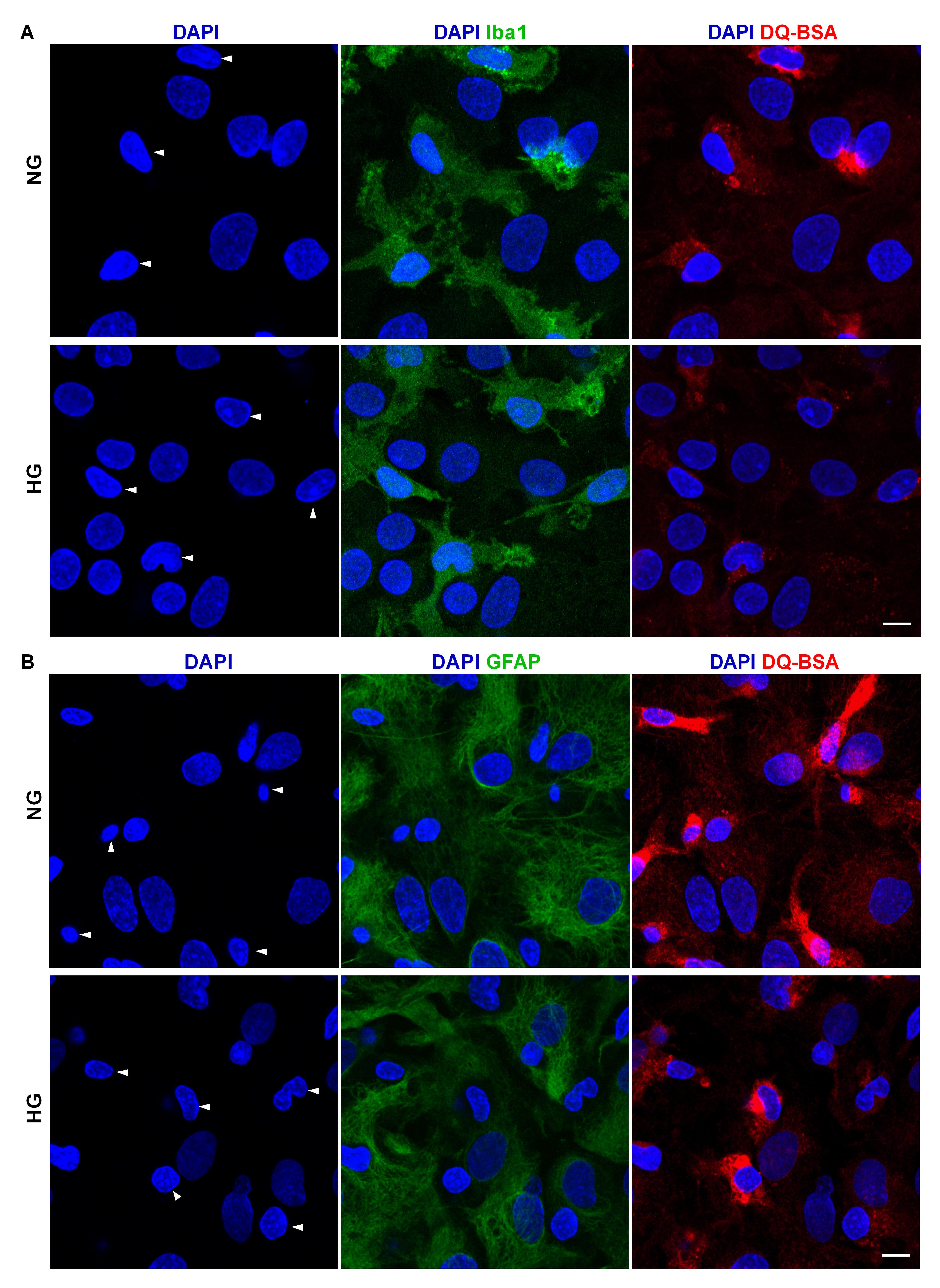

Supplement: S7 Fig — Mixed glia were incubated with DQ-BSA and immunostained with anti-Iba1 or anti-GFAP antibodies. Nuclei were stained using DAPI. (A) Representative confocal images of nuclei of mixed glia (blue, left panel). Merged image of DAPI and immunostaining with anti-Iba1 (green) antibody (middle panel). Merged image of DAPI and DQ-BSA (red, right panel). (B) Representative confocal images of nuclei of mixed glia (blue, left panel). Merged image of DAPI and GFAP immunostaining (green, middle panel). Merged image of DAPI and DQ-BSA (red, right panel). Arrowheads indicate microglia, which have smaller nuclei compared to astrocytes. NG, 5.5 mM glucose-containing media; HG, 25 mM glucose-containing media. Scale bar, 50 μm. (TIF) [file pone.0260966.s007.tif]

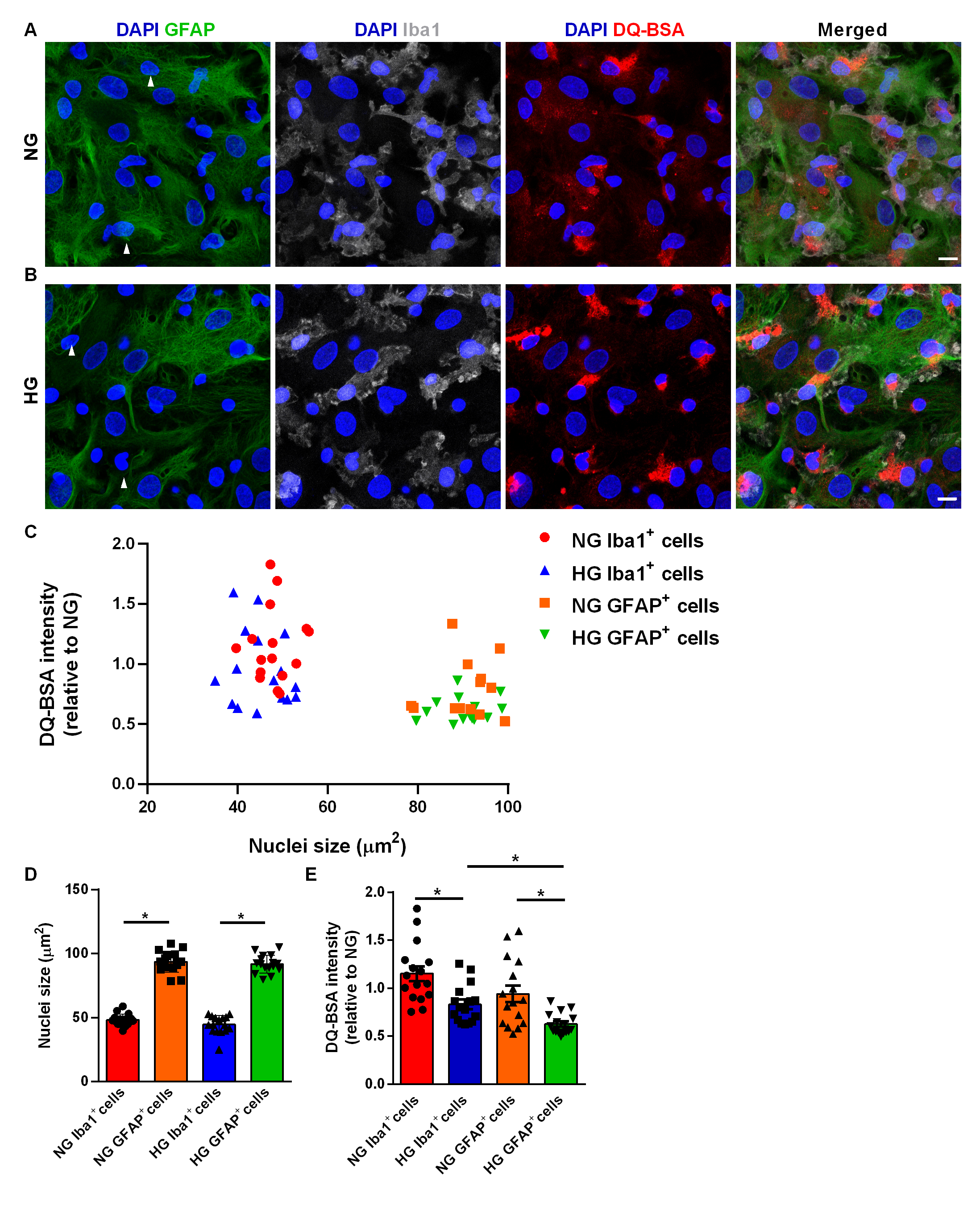

Supplement: S8 Fig — (A, B) Representative confocal images of cleaved DQ-BSA (red) of NG and HG mixed glia cultures simultaneously immunostained with anti-Iba1 (white) and anti-GFAP (green) antibodies. Nuclei were stained using DAPI (blue). (C) The DQ-BSA cleavage activity and nuclei size of Iba I-positive microglia and GFAP-positive astrocytes in NG and HG mixed glia cultures (n = 16). (D) The nuclei size of Iba I-positive microglia and GFAP-positive astrocytes in NG and HG mixed glia cultures. (E) The DQ-BSA cleavage activity of Iba I-positive microglia and GFAP-positive astrocytes in NG and HG mixed glia cultures. Nuclei were stained using DAPI (blue). Arrowheads indicate microglia, which have smaller nuclei compared to astrocytes. NG, 5.5 mM glucose-containing media; HG, 25 mM glucose-containing media. Scale bar, 25 μm. Experiments were repeated at least three times. Data is expressed as the mean ± SEM. Statistical differences between groups were determined by Unpaired Student’s t-test, and are labeled with *(p < 0.05). (TIF) [file pone.0260966.s008.tif]

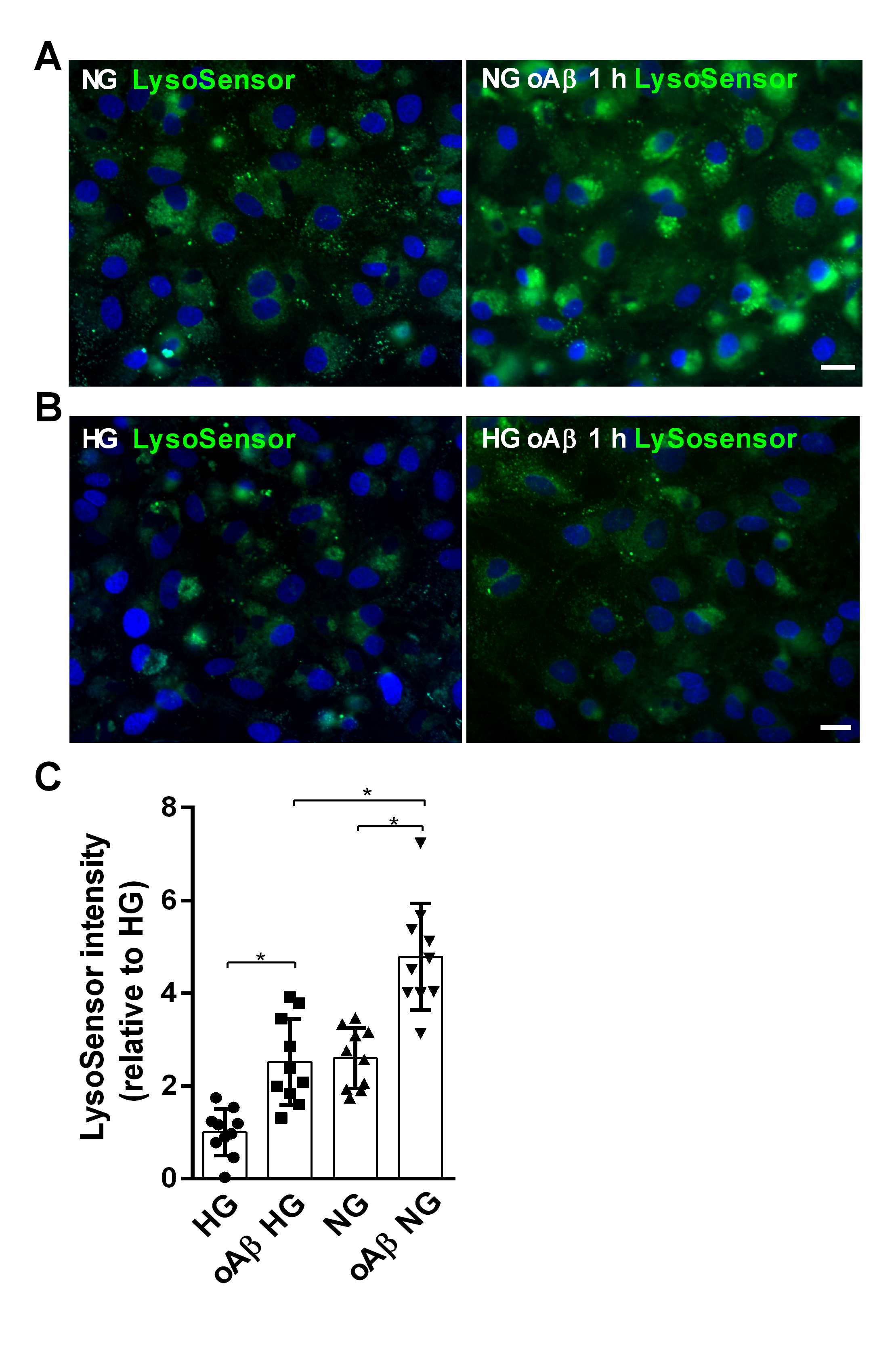

Supplement: S9 Fig — (A, B) Representative LysoSensor fluorescent images of live NG mixed glia and HG mixed glia at DIV16 incubated without treatment (left panel) and with oAβ for 1 h (right panel). (C) Quantification of the relative LysoSensor fluorescence intensity in NG and HG mixed glia incubated with and without oAβ. Nuclei were stained using DAPI (blue). NG, 5.5 mM glucose-containing media; HG, 25 mM glucose-containing media. Scale bar, 20 μm. Experiments were repeated at least three times. Data is expressed as the mean ± SEM. Significant differences between groups were determined by one-way ANOVA followed by Bonferroni post-hoc tests, and are labeled using * (p <0.05). (TIF) [file pone.0260966.s009.tif]

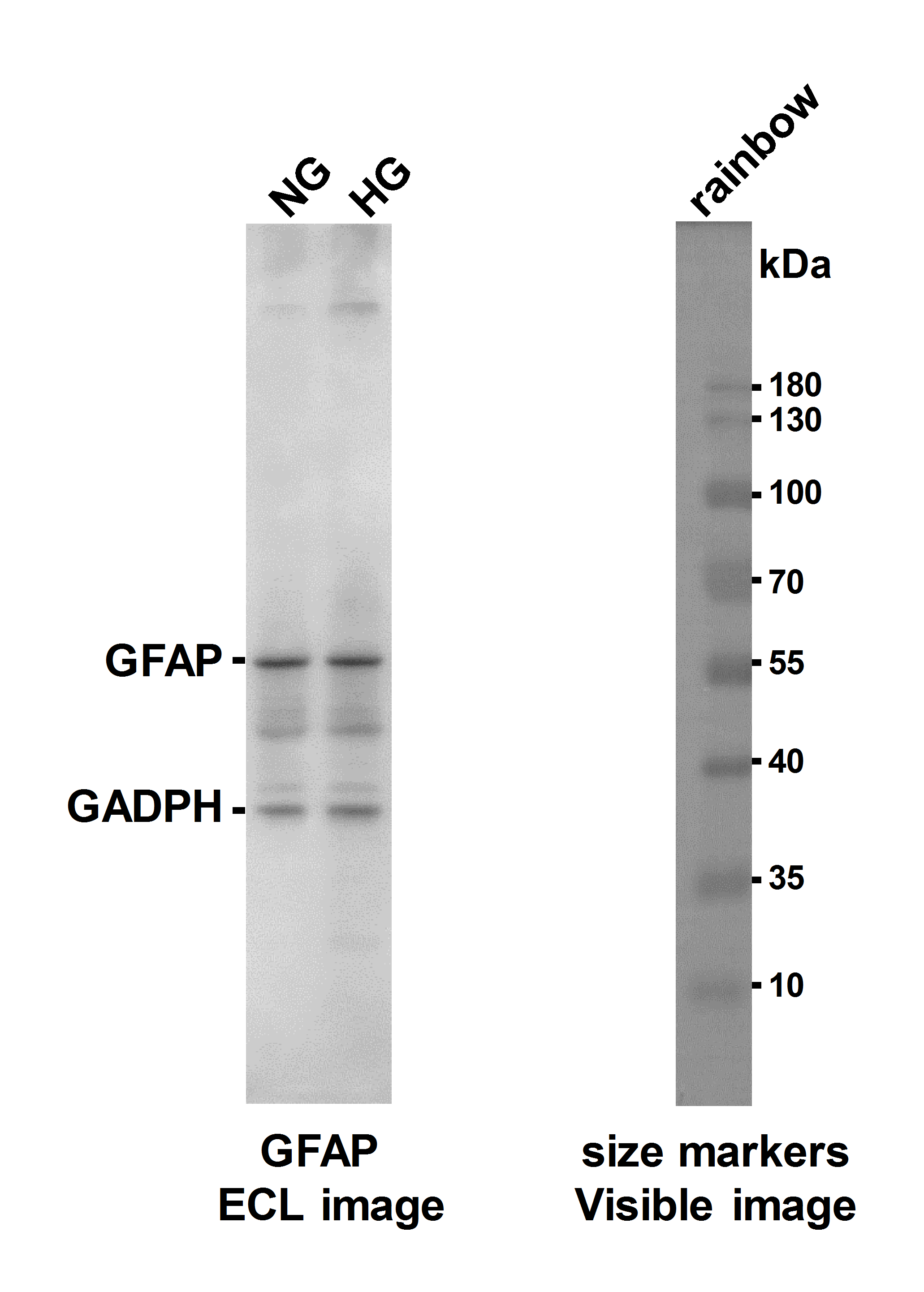

Supplement: S10 Fig — (TIF) [file pone.0260966.s010.tif]

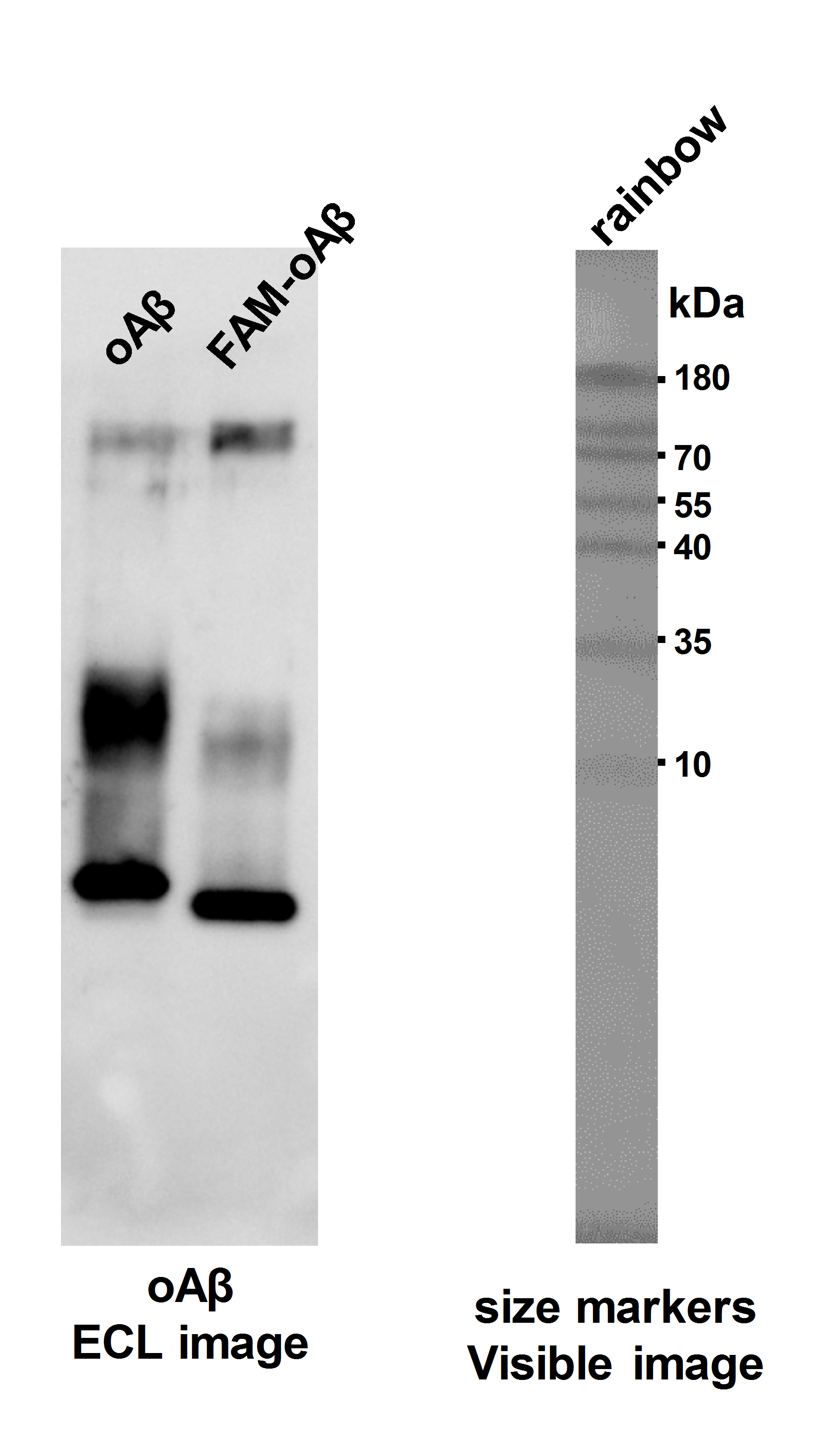

Supplement: S11 Fig — (TIF) [file pone.0260966.s011.tif]

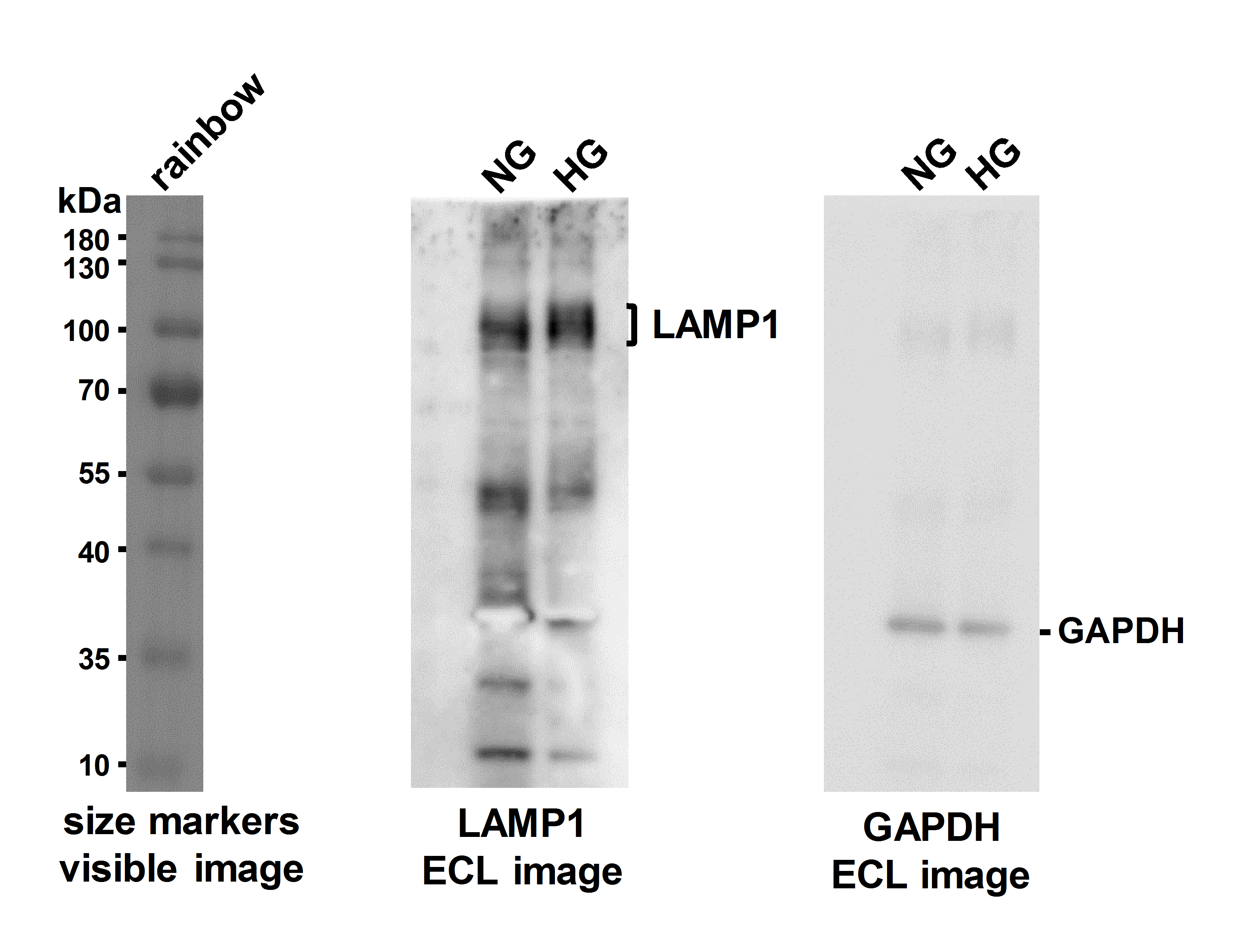

Supplement: S12 Fig — (TIF) [file pone.0260966.s012.tif]

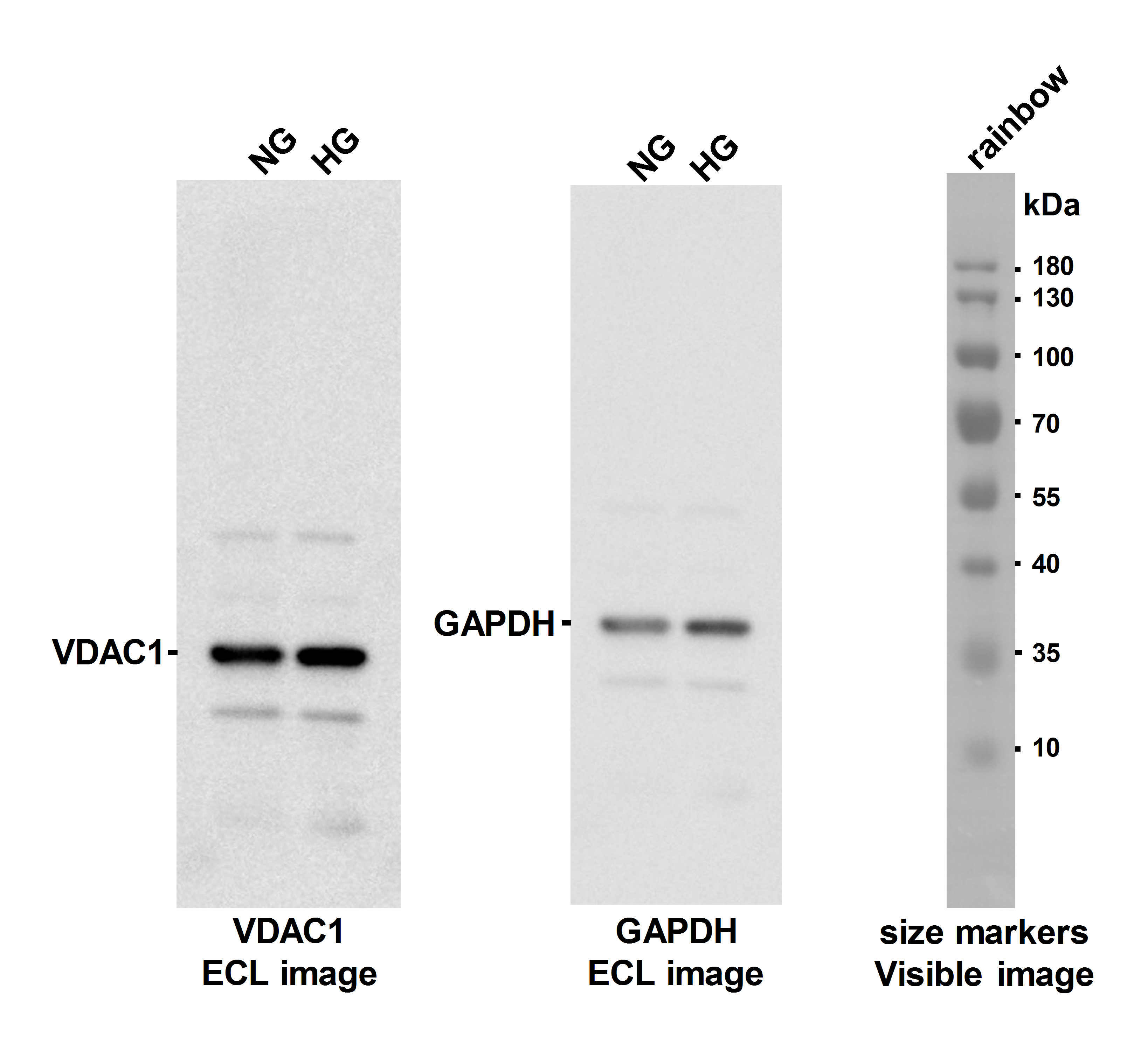

Supplement: S13 Fig — (TIF) [file pone.0260966.s013.tif]
